# Domestication of Transposable Elements into MicroRNA Genes in Plants

**DOI:** 10.1371/journal.pone.0019212

**Published:** 2011-05-03

**Authors:** Yang Li, Chaoqun Li, Jie Xia, Youxin Jin

**Affiliations:** 1 School of Life Sciences, Shanghai University, Shanghai, People's Republic of China; 2 State Key Laboratory of Molecular Biology, Institute of Biochemistry and Cell Biology, Shanghai Institutes for Biological Sciences, Chinese Academy of Sciences, Shanghai, People's Republic of China; 3 Department of Gastroenterology, Shanghai Public Health Clinical Center Affiliated to Fudan University, Shanghai, People's Republic of China; Kyushu Institute of Technology, Japan

## Abstract

Transposable elements (TE) usually take up a substantial portion of eukaryotic genome. Activities of TEs can cause genome instability or gene mutations that are harmful or even disastrous to the host. TEs also contribute to gene and genome evolution at many aspects. Part of miRNA genes in mammals have been found to derive from transposons while convincing evidences are absent for plants. We found that a considerable number of previously annotated plant miRNAs are identical or homologous to transposons (TE-MIR), which include a small number of bona fide miRNA genes that conform to generally accepted plant miRNA annotation rules, and hairpin derived siRNAs likely to be pre-evolved miRNAs. Analysis of these TE-MIRs indicate that transitions from the medium to high copy TEs into miRNA genes may undergo steps such as inverted repeat formation, sequence speciation and adaptation to miRNA biogenesis. We also identified initial target genes of the TE-MIRs, which contain homologous sequences in their CDS as consequence of cognate TE insertions. About one-third of the initial target mRNAs are supported by publicly available degradome sequencing data for TE-MIR sRNA induced cleavages. Targets of the TE-MIRs are biased to non-TE related genes indicating their penchant to acquire cellular functions during evolution. Interestingly, most of these TE insertions span boundaries between coding and non-coding sequences indicating their incorporation into CDS through alteration of splicing or translation start or stop signals. Taken together, our findings suggest that TEs in gene rich regions can form foldbacks in non-coding part of transcripts that may eventually evolve into miRNA genes or be integrated into protein coding sequences to form potential targets in a “temperate” manner. Thus, transposons may supply as resources for the evolution of miRNA-target interactions in plants.

## Introduction

Transposable elements (TEs) are able to mobilize and propagate in the host genomes. Their mobilization can cause catastrophic damage to the host, such as disruption of protein-coding genes, perturbation of gene expression patterns or even large scale genome rearrangement and chromosomal breakage [1]. According to intermediates in the propagation cycle, TEs are classified into retrotransposons (Class I) and DNA transposons (Class II) [2]. Regarding to the ability to mobilize, TEs can be categorized into autonomous TEs, which encode enzymes needed for their own transposition, and non-autonomous TEs whose transposition rely on cognate autonomous TEs [2]. The composition and amount of TEs in the genome is highly specific in different species [1].

Small RNA dependent silencing mechanisms have evolved to harness the activity of the TEs in eukaryotes [3], [4], [5]. In plants and fungi, TEs are silenced by repeat associated siRNAs (rasiRNA). The ∼24-nt rasiRNAs are processed from TE-derived transcripts by dicer-like enzymes and then loaded into RISC (RNA Induced Silencing Complex), in which Argonaute proteins play key roles, to guide DNA methylation and/or repressive histone modification. As a result, the homologous TEs are heterochromatized and therefore transcriptionally inactivated [3], [5]. In metazoan, Piwi Interacting RNAs (piRNA) play a role in TE silencing in the germline. Piwi proteins, another clade of Argonaute proteins, bind piRNAs to induce silencing of TEs via cleavage of transcripts, DNA methylation or histone modification [3], [5]. Various mechanisms have evolved to silence TEs in other species, for example, programmed DNA elimination in *Tetrahymena*, quelling, repeat-induced point mutation (RIP) and meiotic silencing of unpaired DNA (MSUD) in *Neurospora crassa* [3], [5]. In common, the TE silencing relies on the major RNAi components, such as Argonaute proteins, Dicer or Dicer-like enzymes and RNA dependent RNA polymerase (RDRP).

Opposite to the destructive roles, TEs drive the evolution of genes and genomes at many aspects [1]. TEs are known to be able to mediate translocations, gene and segmental duplications leading to gene family expansions that may further undergo selection and diversification. Jumping of the TEs can also cause alteration in gene expression patterns since many of them contain elements for transcriptional regulation and splicing signals [1]. Some of the TEs capture host sequences of cellular genes and form new open reading frames. There are many examples of domestication of TE proteins into functional host proteins, especially the transposases [6], [7]. A considerable amount of DNA binding proteins are derived from transposases encoded by DNA transposons [6]. For example, evidences support that the RAG1 and RAG2 proteins that carry out V(D)J recombination of immunoglobulin genes arise from domestication of DNA transposons protein [8]. Telemerase is evidenced to be tightly linked to the reverse transcriptase of non-LTR retrotransposons [9], [10].

Recent studies have shown that transposons also contribute to the evolution of miRNA genes, products of which are approximately 21-nt small RNAs that can mediate sequence-specific regulation of endogenous gene expressions by binding to complementary sites on target mRNAs [11], [12]. The key components of miRNA pathway are identical or closely related to those in siRNA pathways. Both miRNA and siRNA pathways require dicer or dicer-like enzymes in their biogenesis and Agonaute proteins as major component in effector complex [11], [13]. Homology in key components of siRNA and miRNA pathway suggests that they may have common origin. However, miRNAs have distinct set of features. First, the miRNA precursors are single stranded transcripts that can fold into characteristic hairpin structures, which is transcribed from loci distinct from their target genes. Second, usually each miRNA gene give rise to only one functional RNA duplex formed by miRNA and miRNA* in specific location. Third, miRNAs act in trans to repress cellular gene expression through translational inhibition, slicing or destabilizing target mRNAs when hybridized to their target sites [13]. Instead of acting as defenders like siRNAs (except tasiRNA) or piRNAs, miRNAs participate in the regulation of a wide range of endogenous processes including development, metabolism, stress response etc., and often form regulatory circuits with transcription factors [11], [12], [13]. Due to confusability in distinguishing miRNA from siRNA in plants, a uniformed rule has been made to guide the annotation of plant miRNAs [14].

Several hypotheses have been proposed to explain the origin of miRNA genes. First, evidences from some recently spawned miRNA genes support their genesis by inverted duplication of target genes [15]. Second, the abundant fortuitous foldbacks in the genomes can also supply as a source of miRNA genes [16]. Third, in mammals, some miRNAs were found to be exactly TEs or their derivatives and presence of cognate elements in the 3′ UTR of protein-coding genes may confer susceptibility to miRNA regulation [17], [18], [19], [20], [21]. Similarly, in plants many miRNA-like hairpins, which give rise to small RNAs chemically undistinguishable from miRNAs, are exactly TEs or homologous to TEs, including some annotated miRNAs deposited in miRbase [22]. However, since miRNA and siRNA can not be distinguished by biochemical nature, whether these TEs give rise to bona fide miRNAs is not convincingly evidenced [12]. We searched and characterized the TE-related plant miRNAs deposited in miRbase and analyzed their sRNA producing profile to assess their conformability to updated plant miRNA annotation rules [14]. Furthermore, we analyzed the original target genes of TE-related “miRNAs”, which contain homologous TE sequences in their CDS. Our results support that some plant miRNAs have evolved from TEs and incorporation of cognate TEs into CDS of protein-coding genes may lead to their integration into miRNA regulation network.

## Results

### The previously annotated miRNAs homologous to TEs

**Figure 1:**
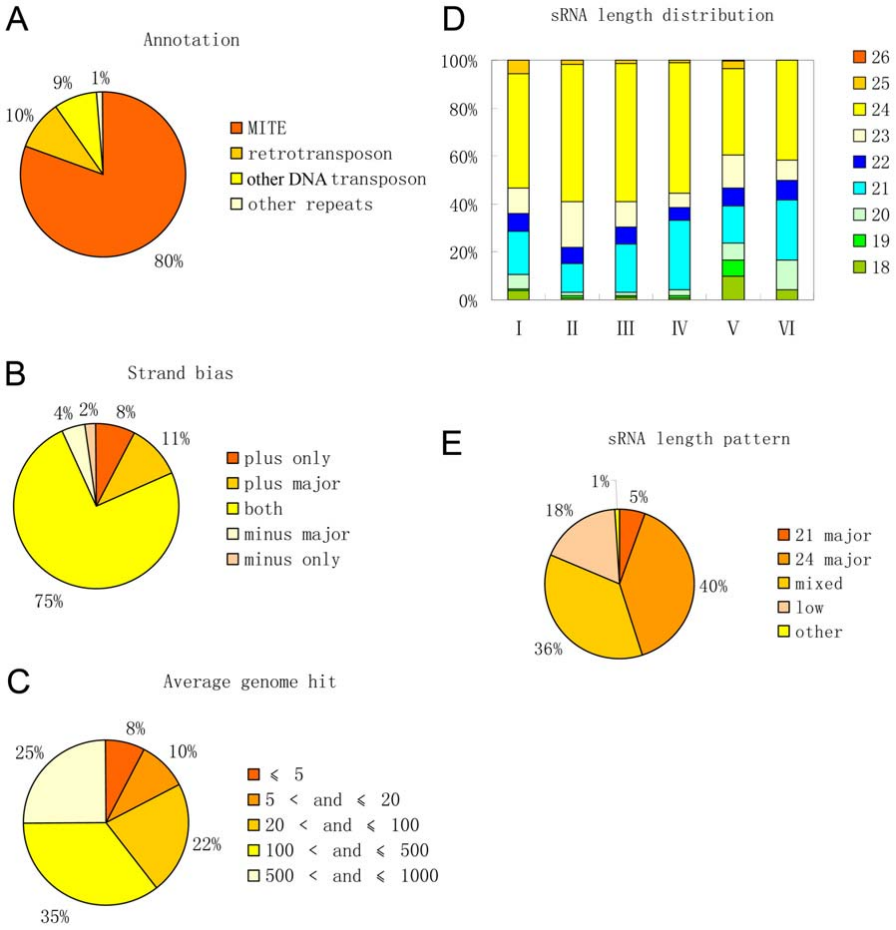
Characterization of annotated rice miRNAs related to repeats. (A) Percent of TE-MIRs with regard to the types of repeats. (B) Strand bias of the sRNAs produced from the hairpins of the TE-MIRs. (C) Average copy number of the sRNAs produced from the TE-MIRs in rice genome. (D) Length distribution of the sRNAs produced from the TE-MIRs in different sequencing databases. I, CSRDB run 1; II, CSRDB run 2; III, GSE11014 solexa part; IV, GSE11014 454 part; V, GSE13152; VI, MyRNA. (E) Percent of TE-MIRs with regard to product length (five types see the text).

miRNA precursors can fold into hairpin structures, which can be processed by dicer or dicer-like enzymes. Numerous such hairpins pervade plant genomes, which give rise to siRNAs. This brings difficulty to distinguish miRNAs from the endogenous hairpin derived siRNAs (hsiRNA) [23]. Some annotated plant miRNAs in the miRbase were found to be TE or TE derivatives [22], [23]. It was believed that this kind of gene is likely to be evolutionary intermediates from TE to miRNA genes [22]. In order to find out whether there are bona fide TE-derived miRNAs in plants, miRNA stem-loop sequences (referred to as MIR thereafter) of seven species (*Arabidopsis thaliana*, *Brassica napus*, *Glycine max*, *Medicago truncatula*, *Oryza sativa*, *Solanum lycopersicum*, *Triticum aestivum*) were BLAST searched against the TIGR Plant Repeat database. We got 106 TE-MIRs (2 in *A.thaliana*, 92 in *O.sativa* and 12 in *T.aestivum*), of which at least one HSP (High-scoring Sequence Pair) suffice E ≤ 0.005 (Table S1). Among them, 30 (2 in *A.thaliana* and 28 in *O.sativa*) were also indentified as TE or TE homologues using a different method in a recent study [22] (Table S1). It was only in rice that we obtained a considerable amount of TE related MIRs (TE-MIR). So following analysis was focused on the rice TE-MIRs. Examination through the genome browser showed that all the 92 rice TE-MIRs overlap fully or partially with TE or other repeats. This further confirmed their TE origins. Among the rice TE-MIRs, 80% are MITE, 10% are retrotransposons and 9% are other DNA transposons (Figure 1A). This is consistent with the previous findings that many MITEs are small RNA generating loci with hairpin structure [22], [24], [25].

### Characterization of the TE-MIRs

The major rule to annotate miRNA in plants is whether precise excision of approximately 21-nt miRNA/miRNA* duplex from qualifying stem-loop can be well documented [14]. We exploited the publicly available small RNA sequencing data to characterize the configuration of the mature products from the TE-MIRs. Small RNA sequences from CSRDB (rice part), MyRNA (rice part), NCBI GEO (GSE11014 and GSE13152) were mapped on the stem-loop sequences of the rice TE-MIRs identified above [26], [27], [28], [29]. Only perfect matches were considered and both strands were considered. Totally, 3143 unique small RNAs were located on the 91 TE-MIRs. We took NCBI GEO (GSE11014 and GSE13152) as main reference for small RNA expression because their sequencing scales are the largest. Regarding to strand orientation, we classified the small RNA expression pattern into five categories: 1) plus only, only the plus strand have matched small RNAs; 2) minus only, only the minus strand have matched small RNAs; 3) plus major, only low frequency sRNAs matched minus strand and negligible compared to plus strand; 4) minus major, same to “plus major” except strand; 5) expressions of small RNAs on both strands are comparable in abundance or dominant sRNA generating strand differ in different databases. It was reported that some annotated miRNA genes generate small RNAs from both strands [29]. Similarly, we found that the majority (68 in 92) of the TE-MIRs showed similar level of small RNA reads matched on both strands (Figure 1B and Table S2). However, a substantial portion (17 in 92) of TE-MIRs generates small RNAs predominantly from the plus strand (Figure 1B and Table S2).

Since TEs often have high copy numbers in the genome, we next examined the exact copy number of the TE-MIR derived small RNAs in the sequenced rice genome. For each TE-MIR, we calculated the average number of genome hit for all the related small RNAs. Most of the TE-MIRs matched small RNAs with medium to high copy numbers significantly exceeding that of the canonical miRNAs (compared to canonical rice miRNAs from miR156 to miR408 in Table S3, p<0.00001) (Figure 1C and Table S2). However a subgroup of the TE-MIRs have improved genome specificity and generate dominantly low copy small RNAs (Table 1 and Figure 1C). The production of small RNAs from both strands is an excluding principle in miRNA annotation because it indicate the biogenesis from double stranded RNAs [14]. As for the high-copy small RNAs, the situation is more complicated because the exact genomic locus that gives rise to the small RNA can not be determined. First, sRNAs from both strands may be processed from dsRNAs either generated by RDR (RNA dependent RNA polymerase) dependent pathways or formed by antisense transcripts of the TE-MIRs. Second, hairpins of antisense copies of the TE-MIRs located elsewhere in the genome may give rise to sRNAs that match minus strand of the corresponding TE-MIRs. Third, sRNAs arising from the perfectly complementary segment of the stem-loop also match the minus strand. Therefore, the biogenesis of the small RNAs can only be inferred for the low copy TE-MIRs. The high copy sRNAs from the TE-MIRs might represent the products of homologous TE groups, possibly with similar stem-loop structures, rather than single genomic locus. In the analysis, we did find a few examples of low copy TE-MIRs from the highly repeated jumping elements.

**Table 1:** Nine TE derived miRNA genes.

MIR	N^a^	Strand^b^	Hit^c^	Len dis^d^	HCDS^e^	TF^f^
osa-MIR812f	12	plus only	3.5	24 major	no	null
osa-MIR812h	40	plus major	9	24 major	yes	yes
osa-MIR812i	29	plus major	7	24 major	yes	no
osa-MIR812j	77	plus major	9	24 major	yes	yes
osa-MIR1848	17	plus only	16	21 major	yes	yes
osa-MIR1850	29	plus major	2	21 major	no	null
osa-MIR1868	58	plus only	1	24 major	yes	yes
osa-MIR1877	25	plus only	1	24 major	no	null
osa-MIR1879	29	plus major	2	24 major	yes	yes

a. Number of small RNAs perfectly aligned to miRNA foldback at both strand.

b. Strand bias of the expression of the sRNAs on each strand.

c. Average number of hit in the Osa1 v6 rice genome of all the small RNAs.

d. Length distribution pattern of the small RNA products.

e. Whether the miRNA foldback is homologous to CDS sequence.

f. Whether any small RNA on the plus strand of the foldback was predicted to target the homologous CDS by TargetFinder at the threshold of score ≤ 4 and MFE ratio ≥ 73.

**Figure 2:**
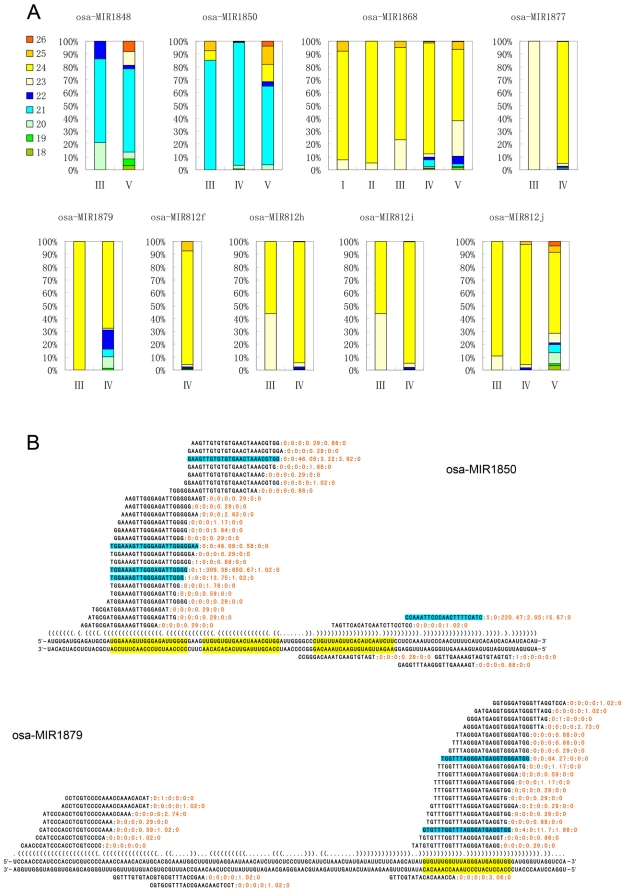
Bona fide TE-derived plant miRNAs. (A) Length distribution of the sRNAs produced from each of the TE derived miRNA hairpins. The small RNA sequencing databases are indexed the same as in Figure 1. (B) Examples of sRNA production of TE derived miRNA hairpins shown as alignments of sRNA products with the miRNA hairpin sequences and predicted secondary structures. Sequencing abundance values are shown at right side of each sequence using six numbers separated by “:”, which are values in different databases: from left to right, CSRDB run 1; CSRDB run 2; GSE11014 solexa part; GSE11014 454 part; GSE13152; MyRNA. sRNAs with significantly higher expressions are highlighted in blue background. The annotated mature miRNA or miRNA* were highlighted in yellow background.

Size can be used as a reference for small RNA classification. The most abundant sRNA populations are 24-nt and 21-nt in angiosperms [30]. Most of miRNAs are 21-nt in length while 24-nt sRNAs are mainly taken up by RDR2 dependent heterochromatic siRNAs that target TEs for silencing [31]. A recent study found that siRNAs from MITEs in *Solanaceae* were primarily 24-nt in length [24]. The biogenesis for some of them requires RDR2, DCL3 and DCL4 [24]. We analyzed the sRNA length distribution of the TE-MIRs both collectively and as individual. In all of the six independent databases, 24-nt sRNAs were the most abundant. Interestingly, in 4 of 6 databases 21-nt sRNAs were the second most abundant (Figure 1D). The percent of 21-nt fraction, approximately 10∼35%, is significantly higher than that of the siRNAs bound by AGO4, which is about 5%, in *A.thaliana* [32]. This suggests that at least part of the TE-MIRs may experience a transition from producing 24-nt to 21-nt sRNAs. To describe the length distribution pattern of the individual TE-MIRs, we categorized the patterns into five types. First, if sequencing reads are less than 10 for all the length classes, the length distribution is difficult to be determined and this type was referred to as “low”. Second, if the sequencing abundance was mainly contributed by 21-nt sRNAs unanimously in all the databases with at least one usable value (≥10), this type was referred to as “21 major”. Third, the “24 major” was used to refer to the same situation as “21 major” except the major population of the sRNAs is 24-nt. Forth, If a clear predominant size population could not be determined due to disagreement among different databases or similar expression level of different sizes, this type was dubbed “mixed”. Finally, if the predominant size population was outside of the range from 20 to 24-nt, this type was called “other”. The classification is based on the finding that the most abundant size populations of angiosperm small RNAs are 21 and-24 nt [30]. “21 major” also include few cases in which the 20 or 22-nt are the predominant population. Similarly, “24 major” also include rare cases that 23-nt predominate. Based on such classification, 36 in 91 of the TE-MIRs produce predominantly 24-nt small RNAs (Table S2). 33 TE-MIRs produce comparable amount of 21-nt and 24-nt small RNAs (Table S2). Of 5 TE-MIRs, the major products are 21-nt sRNAs (Table 1, Figure 1E, Table S2 and Figure 2A). This suggests that some TE-MIRs produce predominantly 21-nt sRNAs like the canonical miRNAs. The heterogeneity in product sizes of the TE-MIRs may reflect the spectrum of evolutionary intermediates from TEs to MIR with respect to different mechanisms in biogenesis.

### The Evolution of TE derived hairpins into miRNA genes

Many previously annotated plant miRNAs were found to be TE related and their annotation as miRNAs were questioned [12], [23]. Therefore, even if some annotated plant miRNAs deposited in the miRbase were proved to have TE origins, evidences are still lacking for the existence of bona fide TE derived miRNAs that match the updated plant miRNA annotation rules. We searched for such candidates among the TE-MIRs that suffice: 1) average genome hit of sRNAs was no more than 20; 2) sRNAs matching the TE-MIRs were purely or predominantly from plus strand. Nine such TE-MIRs were found: osa-MIR1848, osa-MIR1850, osa-MIR1868, osa-MIR1877, osa-MIR1879, osa-MIR812f, osa-MIR812h, osa-MIR812i and osa-MIR812j (Table 1). Examination of the genomic context of these TE-MIRs showed that all of them overlapped fully or partially with TEs. 3 located within introns of coding genes and 6 situated in intergenic regions (Figure S1). Two of them, osa-MIR1848 and osa-MIR1850, produced primarily 21-nt sRNAs while the remaining generated 24-nt sRNAs as major products (Figure 2 and Figure S2). All of the 9 TE-MIRs showed the pattern that the sRNA reads concentrated in specific regions, usually two that could form a duplex within the stem (Figure 2B and Figure S2). At least one major sRNA species could be recognized whose abundance was much higher than other weakly expressed variants (Figure 2B and Figure S2). Variants with significant values were also present in a few cases, for example of osa-MIR1850 (Figure 2B). This type is similar to the MIR159/319 family, of which precursor stem-loop give rise to multiple mature miRNAs [33], [34], [35]. The miR* supported by significant sequencing values were observed for osa-MIR1848, osa-MIR1850, osa-MIR1868 and osa-MIR1877 (Figure 2 and Figure S2). For all the nine TE-MIRs, except osa-MIR812h and osa-MIR812i, a series of weakly expressed sRNAs are processed from the region corresponding to the putative miR* (Figure 2B and Figure S2). Rigorously, at least the osa-MIR812f, osa-MIR1848, osa-MIR1868 and osa-MIR1877 suffice the requirements for miRNA annotation. For the other 5 TE-MIRs, precise excision of the putative miR\miR* were supported by the sequencing data in spite of scarce sRNA reads from minus strand. These 5 are also likely to be bona fide miRNA genes. We referred to these 9 TE-MIRs as typical TE-MIRs and other 83 as atypical TE-MIRs. All these 9 typical TE-MIRs evolved from various types of TEs, seven from MITE, one from retrotransposon and one from a kind of unclassified transposon (Figure S1). Notably, in the osa-MIR812 family, four of them have evolved into typical TE-MIRs while the other 6 keep the atypical features such as high copy, smeared excision etc. (Table 1 and Table S2).

### Formation of foldback structures by juxtaposition of two inverted TE copies

**Figure 3:**
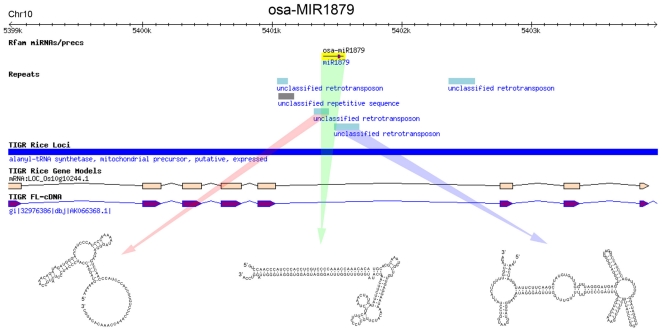
Formation of miRNA hairpin by juxtaposition of two cognate TEs in inverted orientations. Genomic context with annotation information was shown for the TE-MIR locus. Predicted secondary structures corresponding to the segments are indicated by arrows.

The first step for the genesis of novel miRNA genes might be the formation of inverted repeats that can fold into stem-loop structures. Inverted duplication from target genes was proposed to be a possible mechanism [15]. It was also hypothesized that numerous non-autonomous derivatives of autonomous TEs, typically MITEs, can supply as source for new miRNA genes because of their palindromic nature [16]. Interestingly, several human miRNA hairpins were found to be formed by two adjacent inverted LINE2 elements [17], [20]. Such “Complementary Insertions” were also observed for a *Solanaceae* MITE [24]. We also found some similar cases for rice TE-MIRs. The osa-MIR815b and osa-MIR815c hairpins spanned two juxtaposed transposons (Figure S3A and B). Neither of the two adjacent transposons formed qualifying stem-loop structure. However, each contributed one arm to a typical stem-loop structure (Figure S3A and B). However, sequencing data do not support precise excision of miRNA/miRNA* from their stem-loops. Therefore osa-MIR815b and osa-MIR815c are not bona fide miRNAs but possibly pre-evolved miRNAs. The osa-MIR1879 locus, which is a TE derived bona fide miRNA, has similar structure (Figure 3). The two TEs, which are pieced together into osa-MIR1879, are short non-autonomous retrotransposons (Figure 3). These observations indicate that juxtaposition of two inverted TE copies could be an alternative for TE derived hairpin formation. Preferential insertion of multiple copies of cognate TEs with different orientation into one “hot spot” may result in the formation of such locus. Similarly, the osa-MIR1442 locus assembled in the same way. However, the osa-MIR1442 does not have qualifying stem-loop structure. Instead, the two MITEs sharing the osa-MIR1442 have hairpin structures (Figure S3C). Thus the real precursor of sRNAs from this locus may not be the annotated osa-MIR1442 but the two MITE hairpins. This suggests that considering more flanking sequences may improve the accuracy of precursor structure evaluation for the annotation of hairpin derived siRNAs and miRNAs.

### Initial targets of the TE related miRNAs

**Figure 4:**
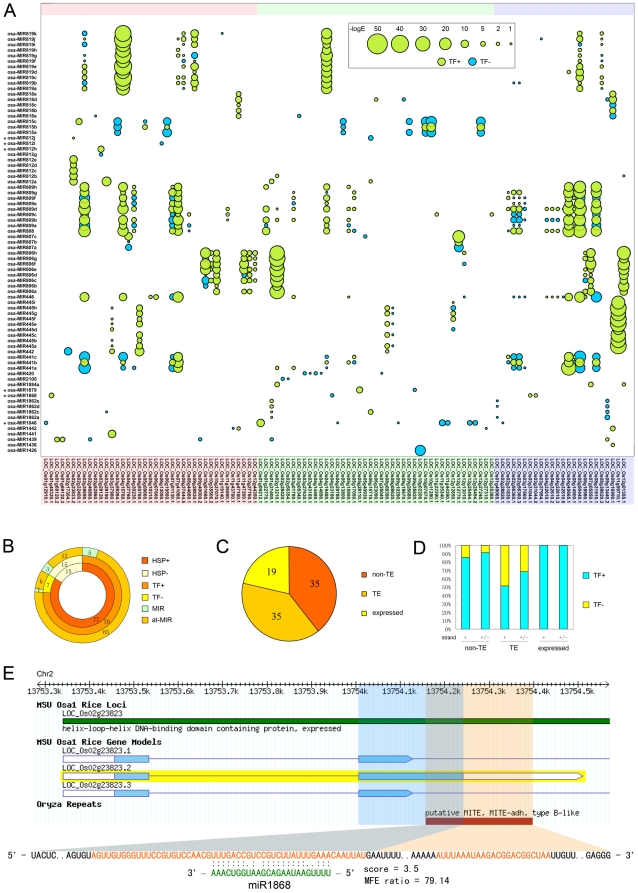
Formation of initial targets of the TE-MIRs by incorporation of cognate TEs into CDS. (A) Bubble plot showing interactions of TE-MIRs with their initial targets, which are protein-coding genes containing segments homologous to TE-MIRs in the CDS. Vertical axis is the TE-MIRs while horizontal axis indicates their initial targets. Bubbles indicate HSPs of TE-MIRs and CDS of protein-coding genes detected by BLAST. Bubble size is proportional with the –logE of the HSP. TF+: at least one TE-MIR small RNA can target related CDS by the prediction of TargetFinder. TF-: CDS was not predicted as target of any small RNA derived from related TE-MIR. Three classes of genes are indicated by different colored backgrounds: Pink, non-TE related proteins; Green, TE-related proteins; Purple, expressed proteins without clear annotation. (B) Summary of initial target search for the TE-MIRs. HSP+, significant similarity with CDS was detected; HSP-, no homologous CDS was detected. TF+ and TF-, the same as in (A). MIR, bona fide miRNA genes listed in Table 1.; at-MIR, TE-MIRs not identified as bona fide miRNA genes. (C) Number of initial target genes of the three classes, non-TE related, TE-related and expressed proteins without clear annotation. (D) Percent of TargetFinder predicted initial targets. TF+ and TF-, the same as in A. +, only small RNAs from plus strand of TE-MIRs were considered; +/-, small RNAs from both strands were considered. (E) A typical example of initial target genes. Genome annotation information is shown in upper part. The second exon's CDS of the splicing isoform 2 is highlighted in blue while the MITE in orange. The region shared by the CDS and MITE is highlighted in grey. Sequence of the MITE is shown in lower part, within which sequence of potential complementarity with small RNAs derived from TE-MIRs are highlighted in orange. Base-pairings with miRNA is shown along with predicted pairing score and MFE ratio.

Plant miRNAs achieve their functionality primarily via inducing site specific cleavage of target mRNAs [13]. Translational repression has also been documented to be an important mechanism [36], [37]. Both depend on the recognition of target gene by perfect or near-perfect base pairings [12]. There are extensive complementarity between young miRNA genes and related target genes detectable by pairwise sequence similarity search tools such as BLAST [11], [15], [29]. This type of target genes may be the initial targets of new born miRNA gene. In order to test whether the TE-MIRs were integrated into endogenous gene regulation networks, we searched the initial targets of the TE-MIRs. Since most target sites of plant miRNAs locate within CDS of protein coding genes [38], [39], we used the TE-MIR sequences to BLAST against the CDS sequences of annotated rice genes. At the threshold of E ≤ 0.05, 509 HSPs (High-scoring Sequence Pair) were retrieved for 77 TE-MIRs and 106 non-hypothetical protein coding genes (including alternative splicing isoforms) (Figure 4A, B). Among those protein coding sequences, 89 were present in at least one HSP with an E ≤ 0.01 (Figure 4C and Table S4). This group might be the initial targets of the TE-MIRs. In the 89 genes, 35 (39.5%) encode TE related proteins, 35 (39.5%) encode annotated cellular proteins and 19 (20%) encode unknown expressed proteins (Figure 4C).

In order to test whether the homologous CDS detected by BLAST search can be potentially targeted by sRNAs generated from the TE-MIRs, we predicted the sRNA-target pairs by TargetFinder at standard thresholds: Score ≤ 4, MFE ratio ≥ 73% [40]. Totally 347 HSPs (68%) were also predicted to be MIR-target pairs that the CDS can be targeted by the sRNAs arising from the homologous TE-MIRs (Figure 4A). We refer to those HSPs as MTPH (MIR-Target Pair HSPs). As for the three functional classes of original targets, 30 in 35 (86%) of non-TE CDS were present in MTPH, 18 in 35 (51%) for TE related CDS and all the 19 (100%) CDS of expressed unknown protein (Figure 4D and Table S4). The MTPH ratio for the TE related genes was obviously lower than the other two indicative of reduced proclivities for TE targeting of the TE-MIRs. When sRNAs from both strands were considered, the MTPH number increased only slightly, 2 more targeted for non-TE and 6 for TE CDS (Figure 4D). This suggests that the sRNAs from plus strand contributed most of the targeting. With respect to the TE-MIRs, 15 did not share significant similarity with rice CDS of non-hypothetical proteins (Figure 4B). Three typical MIRs, osa-MIR812f, osa-MIR1850 and osa-MIR1877, were in this group (Figure 4B). In the 77 TE-MIRs with identifiable putative original targets, 6 were typical MIRs (Figure 4A and B). However, one of them, osa-MIR812i, has lost pairing potential with its initial targets by our prediction (Figure 4A and B). In a dynamic view, homology and sRNA regulation between the TE-MIRs and related CDS can be reflected in a matrix (Figure 4A). In summary, 72 TE-MIRs (94%) formed HSPs with multiple CDS and 67 CDS (75%) with multiple TE-MIRs. The interactions, which include sequence similarity and sRNA targeting between the TE-MIRs and associated CDS, make up complex relationships that may serve as resources for selection. Variations of homology and regulation profile within TE-MIR families can be seen. In many cases, members of the same TE-MIR families diverged to have distinct gene-interacting profiles thus improving complexity of the network further (Figure 4A). These observations suggest that the original interactions of TE-MIR and targets established by cognate TE insertions may have experienced active evolution.

Question arises that how the CDS acquired the TE sequences. A recent study showed that the N gene of *N.glutinosa* integrated the sequence of a MITE into the CDS of an alternative splicing isoform, which is resulted from insertion of the MITE into the third intron of the N gene [24]. Examination of the original targets in rice genome browser showed that 86 in 89 (96.6%) had cognate TE insertions overlapping with its CDS as exemplified by Os02g23823.2, which could form MTPH with osa-MIR1868 derived small RNAs (Figure 4E). The two sRNA complementary regions locate within the MITE insertion. The major one, which is longer and contain a binding site of the most abundant sRNA from osa-MIR1868, situated within the region shared by the CDS and the MITE (Figure 4E). For the other two isoforms, the target-site-containing MITE insertion lay in their introns. This suggests that TE insertion into the protein coding genes may lead to generation of new isoforms possibly via alteration of splicing signal, thus as a result conferring sRNA regulation to the host gene. Interestingly, all the observed insertions were not fully embedded in the CDS but partially overlapped with the CDS. This indicates that the incorporations of TE sequences into the CDS are likely formed through alteration of mRNA splicing or start/stop signal of translation.

### The TE related miRNAs can induce site-specific cleavages of the initial targets

**Figure 5:**
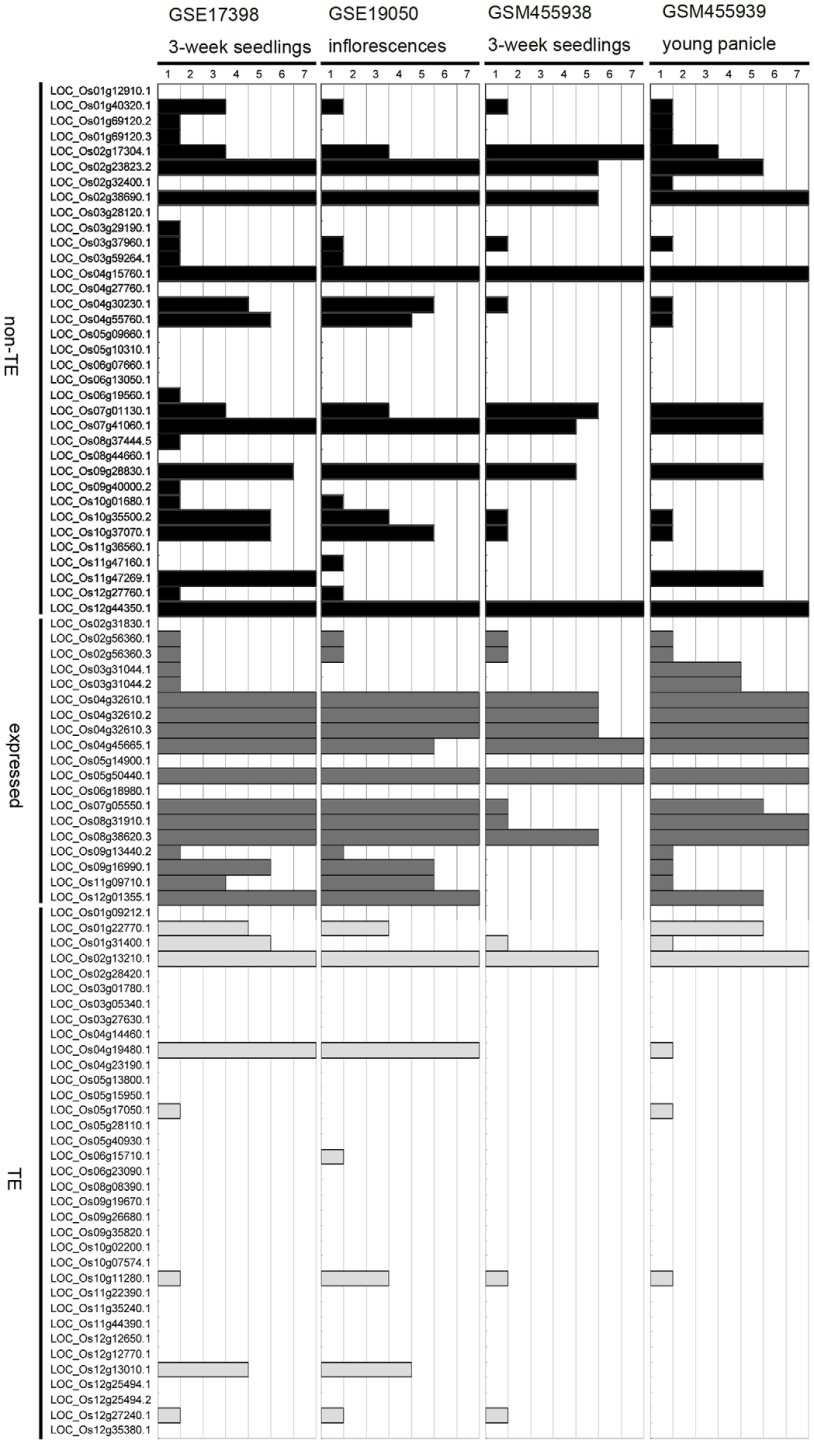
Site-specific cleavages of the initial targets induced by the TE-MIR sRNAs. Cleavages of the initial targets (vertical axis) detected by CleaveLand are described by a score (1 to 7, see text) indicated by horizontal bars. Database of degradome sequencing tags and sampled tissues are indicated at top. Three classes of the initial targets are shown on the left with the same abbreviations as in Figure 4D. Correspondingly, the score bars are shown in black (non-TE), grey (expressed) and light grey (TE) for the three annotation classes.

The major mechanism for miRNA induced gene silencing in plants is site-specific cleavage of the target mRNA at the position complementary to the tenth nucleotide from the 5′ end of the miRNA [12], [13]. Recently, large scale degradome sequencing has been developed to globally identify plant miRNA targets [41], [42], [43], [44], [45]. In order to test whether the TE-MIR derived sRNAs can induce site-specific mRNA cleavages of their initial targets like the canonical miRNAs, we used publicly available rice degradome sequencing data and CleaveLand to detect the specificity of the TE-MIR sRNA-target pairings to the 5′ end of the cleaved remnants [43], [44], [45], [46]. We considered only sRNA-target pairs with a score smaller or equal to 4. Three categories of cleavage profiles are defined by Addo-quaye et, al. [41]. For a target, multiple cleavages of different categories can be detected because many sRNAs can bind to the same target mRNA. We assign a score to summarize the detected cleavages. If at least one cleavage in each of the three categories is detected, the score is 7. If only cleavages of category 1 and 2 are detected, the score is 6. By analogy, category 1 and 3 scored 5; score 4 for only category 1; score 3 for category 2 and 3; score 2 for only category 2; score 1 for only category 3. Based on such scoring, the degradome data of different tissues and from independent studies support very similar profiles of TE-MIR induced cleavages for the initial targets (Figure 5) [43], [44], [45]. 31 of 89 initial targets have at least one category 1 cleavage (Figure 5). In detail, 13 of 35 for non-TE related genes; 13 of 19 for expressed genes; 5 of 35 for TE-related genes (Figure 5). In consensus, transposon-related initial targets have considerably less amount of supported cleavage cases compared to non-transposon or expressed proteins (Figure 5). This is consistent with the results predicted by targetfinder (Figure 4D). Examples of the degradome sequencing data supported cleavages are shown in Figure S4.

## Discussion

Accumulating evidences from studies in animals support the genesis of a substantial portion of miRNA genes from TEs and other genomic repeats [17], [19], [20], [21]. From a considerable amount of TE-MIRs, we identified a small number of bona fide miRNAs that suffice the current plant miRNA annotation rules [14]. Our findings suggest that some plant miRNAs evolve from TEs. However, question arises how the TEs are domesticated from the epigenetically silenced parasites into regulatory genes useful to the host. Previously, a model was proposed to explain the hypothetical transitions from autonomous DNA TEs to miRNA genes via MITEs [22]. Characterizations of the TE-MIRs, which are likely to be evolutionary intermediates from TEs to miRNA genes, enabled us to obtain more information about such transitions.

**Figure 6:**
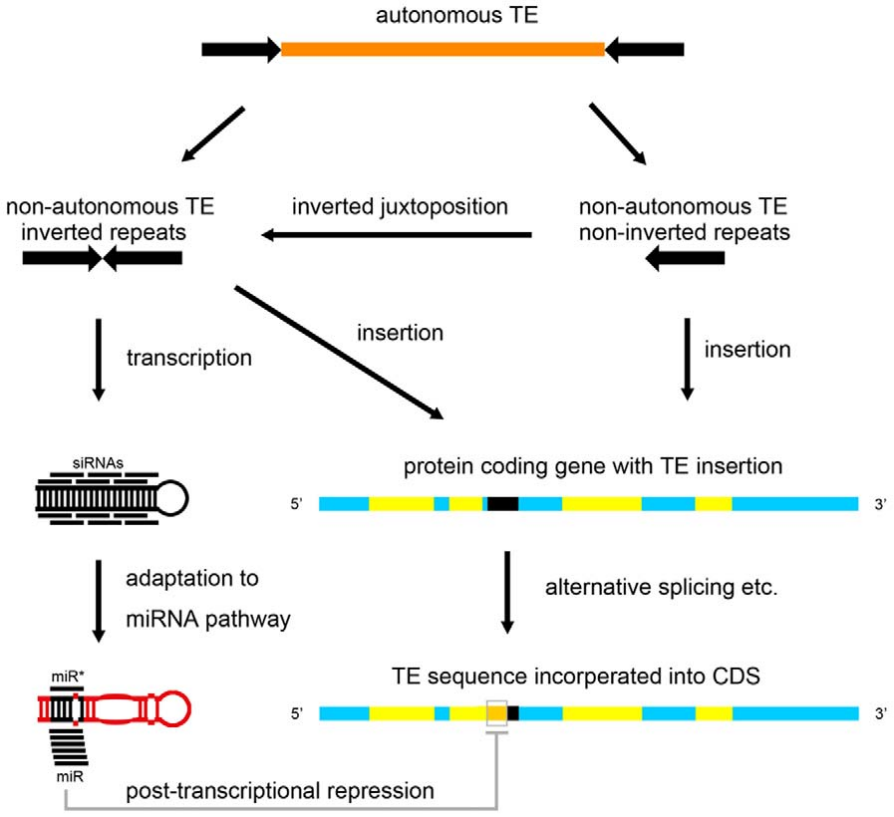
Model for the integration of TEs into miRNA regulation network in plants. Numerous short non-autonomous TEs could be derived from long autonomous TEs. Mobilization of the non-autonomous TEs into actively transcribed units may have two kinds of impact on miRNA evolution. First, inverted repeats of TE or TE derivatives in non-coding transcripts may evolve into miRNA genes. Second, if cognate TE sequences are incorporated into protein coding genes, the miRNA regulated gene expression may begin to evolve. Long perfect hairpins of the proto-MIRs could derive from non-autonomous TEs with inverted repeats or two adjacent short cognate TEs in inverted orientations. Cognate TE insertions adjacent to coding sequences might be incorporated into the CDS through alteration in splicing or sart/stop signal of translation. The proto-MIR and their interactions with corresponding initial targets will be subjected to natural selection. In this way, TEs may supply as resource for the network of miRNA mediated gene regulation in plants.

A critical step for the birth of miRNA genes might be the formation of inverted repeats that can be transcribed into hairpin structured RNAs processable by Dicer-like enzymes. Actively transcribed MITEs with extensive self-complementarities are likely to become pre-evolved miRNA genes [22] (Figure 6) . Indeed, most of the TE-MIRs identified in our analysis are MITEs (Figure 1A). Similarly, we also found TE-MIRs of other types, most of which are short non-autonomous TEs. Alternatively, the original hairpins could also be formed by juxtaposition of two inverted copies of cognate TEs as exemplified by both typical and atypical TE-MIRs and examples from other studies [17], [20], [24] (Figure 3, Figure 6 and Figure S3). Notably, the non-autonomous TEs can not mobilize by themselves. No more trouble for the host will be caused as long as their activators are silenced. Without factors required for mobilization the non-autonomous TEs could be harmless to the host. Consistent with this notion, it was observed that MITE insertions preferentially occurred in genic regions [47], [48]. If such inverted repeats are formed within non-coding transcripts or introns of protein coding genes transcribed by RNA Pol II, they can be transformed into RNA hairpins that may enter hairpin small RNA pathways, which is unlikely to be heterochromatic siRNA pathway because heterochromatic siRNAs depend on Pol IV and Pol V in their biogenesis [3], [4], [5] (Figure 6). As expected, most of the TE-MIRs in our study located in gene rich regions. These TE insertions could be target of epigenetic silencing induced by siRNAs derived from homologous TEs [49]. However, accumulation of point mutations, thus loosing homology with similar repeats, may relieve the TE-MIRs from heterochromatic silencing and improve the genome specificity of the sRNAs liberated from the TE-MIRs. This is supported by the observed gradient of average genome hits of TE-MIR derived sRNAs ranging from 1 to nearly 1000 (Figure 1C and Table S2). Among the TE-MIRs, a few low copy TE-MIRs were identified (Figure 1C, Table 1).

In our analysis, for either individual TE-MIR or as a whole, the 21-nt sRNA took up a substantial portion more than that of the 21-nt fraction in AGO4 bound siRNAs [32] (Figure 1D, E and Table S2). A considerable amount of TE-MIRs give rise to sRNA populations with different sizes (Figure 1E and Table S2). The length distribution of sRNA products may reflect heterogeneity in the processing of the TE-MIRs. This might be attributed to the transition of some TEs from heterochromatic siRNAs pathway, which acts mainly via 24-nt siRNAs, to hairpin siRNA or miRNA pathway, which acts mainly via 21-nt sRNAs [11]. There are three possible explanations. First, a single hairpin-encoding TE locus may follow multiple pathways in a competitive manner. It has been shown that the 21∼22-nt and over 30-nt sRNAs appeared in DCL3 RNAi lines of *N.tabacum* concomitant with the decrease of 24-nt sRNAs when a MITE sRNA was probed by northern blot [24]. Hypothesis of such “dual coding” hairpins was also proposed [22]. Second, young miRNA genes generate heterogeneous populations of small RNAs processed by siRNA biogenesis factors [50], [51]. Observations from sRNA production of TE-MIRs suggest that hairpins derived from TEs possess features of sRNA biogenesis resembling young miRNAs. Third, during evolution, homologous TEs at different loci might enter different pathways. The TE-MIRs located in RNA Pol II transcription unit may enter hairpin siRNA pathway while their homologues may still undergo the heterochromatic siRNA pathway. Taken together, the heterogeneous product size might reflect the transitive state from siRNA to miRNA pathway. The variation in sRNA production with respect to size populations may correspond to various extents of adaptation to miRNA biogenesis of the TE-MIRs.

Once a TE-derived inverted repeat entered the siRNA pathway, a multitude of siRNAs can be processed from the hairpin. The precise excision of the miRNA/miRNA* duplex might be the result of adaptation to miRNA processing and interactions with target genes are similar to the young miRNA genes found in *Arabidopsis* [15] (Figure 6). For the pre-evolved miRNAs, the processing could be imprecise, which produce smeared sRNA products across hairpin arms like siRNAs [15] (Figure 6). From sRNA sequencing databases, we identified only 9 TE-MIRs with precise excision of mature miRNA from the stem-loops (Table 1, Figure 2 and Figure S2). However, the medium to high copy sRNAs from the TE-MIRs may obscure their exact source and make it difficult to determine whether these stem-loops are precisely excised at specific location like canonical miRNAs. Therefore, the number of bona fide TE derived miRNAs might be more than we have identified. A feature of young miRNA genes in *Arabidopsis* is that their foldback arms have extensive complementarity with their initial targets [11], [15], [50]. Indeed, we found that most of the TE-MIRs have significantly identifiable complementarity with protein-coding sequences, including 6 typical TE-MIRs (Figure 4). Our observations suggest that the evolution of TE-MIRs in rice is quite similar to the young miRNA genes in *Arabidopsis*.

Insertion into protein coding genes by cognate TEs may lead to the formation of target genes if the inserted elements are incorporated into exons. This scenario is similar to that observed in mammals. Alu or LINE2 derived miRNA genes might target protein coding genes that carry cognate elements in their 3′ UTRs [17], [20], [21], [52]. In contrast, most of plant miRNA target sites locate within CDS of protein coding genes [12], [13], [23]. The mutagenic effect caused by TE insertion into CDS may ruin functional genes. It has been shown that insertions of TEs into exons are generally deleterious and can be rapidly eliminated in natural selection [53], [54], [55]. Compared to introns, MITEs are significantly underrepresented in exons in rice [56]. However, protein coding genes may capture TE sequences in a more temperate manner. We found that almost all the initial target genes of TE-MIRs contain TE insertions overlapping with CDS. In addition, those TE insertions span the boundaries of CDS and non-coding sequences, which is unlikely to be the result of direct insertion into CDS. These observations suggest that protein coding genes may acquire TE sequences through alteration in splicing and/or initiation or termination signal of translation introduced by non-CDS insertions (Figure 6). This could occur because TEs often contain regulatory elements and in many cases are exonized when inserted into genes [6], [57], [58], [59].

Since the young miRNA genes are usually weakly expressed compared to highly evolved miRNAs, they may not effectively repress target genes. As it was shown that many predicted target genes of young miRNAs were difficult to validate experimentally [14], [23]. However, they might serve as sources of selection for robust, steady, fundamental miRNA regulations. We tested whether these TE-MIRs could induce site-specific cleavages of the initial targets. A substantial number of the initial targets are most frequently cleaved at the central part of the site complementary to TE-MIR sRNAs (Figure 5 and Figure S4). This suggests that the TE-MIR could affect the expressions of the initial targets similar to the miRNAs. As TEs often have numerous copies in the genome, TE-MIRs in one family may target similar group of genes with cognate insertions. Likewise, incorporation of one type of TE into different genes will expand the target spectrum. During evolution the interactions of TE-MIRs and initial targets may rearrange via changes in sRNA expression and their complementarity with targets. Advantageous interactions might be selected from a myriad of initial interactions. The dynamic interactions of rice TE-MIRs and their initial targets during the evolution could be viewed in a matrix (Figure 4A). The TargetFinder supported targeting rate of TE related proteins was lower than non-TE related proteins indicative of functional shift from TE silencing to cellular gene regulation for the TE-MIRs (Figure 4C). This is further supported by degradome sequencing data that cleavages induced by TE-MIR sRNAs are strongly biased to non-TE related genes (Figure 5). Notably, the “cognate TE insertion” model resembles the “inverted duplication of target gene” model in the expected complementarity between young miRNAs and their targets [15]. However, “cognate TE insertion” could result in many different groups of initial target genes for the TE-MIRs, while miRNAs generated from inverted duplication of target gene have purely one group of target genes.

Taken together, our results suggest that plant TEs can evolve into new miRNA genes. Incorporation of homologous TE sequences into protein-coding sequences could result in the formation of target genes. Through formation of miRNA and target genes, TEs could supply as resource for the network of miRNA mediated regulation of gene expression in plants. In addition to the previously reported roles of TEs in host genome evolution, the domestication of TEs into miRNA regulation system could be another important contribution to the host in plants.

## Materials and Methods

### Identification of TE related and annotated miRNAs

miRNA foldback sequences were downloaded from miRbase (version 13.0) [60]. Then BLAST (version 2.2.14) searched against repeat sequences of corresponding species in TIGR Plant Repeat database (ftp://ftp.tigr.org/pub/data/TIGR_Plant_Repeats) [61]. Overlapping of the miRNAs with repeat sequences in the rice genome was examined manually through rice genome browser (http://sundarlab.ucdavis.edu/cgi-bin/smrna_browse/rice2).

### Expression analysis using existing small RNA sequencing data

Rice small RNA sequences were downloaded from CSRDB, MyRNA and NCBI GEO (GSE11014 and GSE13152) [26], [27], [28], [29]. Sequencing abundances of the small RNAs were normalized to transcripts per quarter million (TPQ) for GSE13152. Sequencing values from CSDRB, MyRNA and GSE11014 were used directly without further processing. The small RNAs were mapped onto the miRNA foldback sequences without any mismatch or indel by a costumed perl script. Genome hit number of the small RNAs were obtained by mapping the short sequences to rice genome (J Craig Venter Institute, osa1 version 6.0, downloaded from ftp://ftp.plantbiology.msu.edu/pub/data/Eukaryotic_Projects/o_sativa/annotation_dbs/pseudomolecules) using oligomap [62].

### Search of initial targets for the TE related miRNA or hairpin siRNAs

The repeat associated miRNA foldbacks in rice were BLAST searched against the CDS of the J Craig Venter Institute rice annotation database (osa1 version 6.0, downloaded from ftp://ftp.plantbiology.msu.edu/pub/data/Eukaryotic_Projects/o_sativa/annotation_dbs/pseudomolecules). High-scoring Sequence Pairs (HSP) with an E ≤ 0.05 and genes having at least one HSP with an E ≤ 0.01 were considered. Genes encoding hypothetical proteins were removed. Then, CDS of the resulted genes were used for target gene prediction of small RNAs derived from cognate miRNA foldbacks using TargetFinder [40]. MFE ratios were calculated based on folding energy predicted using RNAduplex in Vienna RNA Secondary Structure Package (version 1.8) [63]. Genomic annotation information of the target genes were manually inspected through the Rice Genome Browser (URL: http://rice.plantbiology.msu.edu/cgi-bin/gbrowse/rice/).

### Detection of site-specific cleavages for miRNA targets

Rice degradome sequencing data were downloaded from NCBI GEO (GSE17398, GSE19050 and GSE18248) [43], [44], [45]. The software CleaveLand was downloaded from http://homes.bio.psu.edu/people/faculty/Axtell/AxtellLab/Software.html [46]. Sequencing tags that can be mapped on the cDNAs of the targets along with the initial target cDNA and TE-MIR derived sRNAs are used for CleaveLand analysis. The cDNA sequences used in CleaveLand analysis are the same as that used for TargetFinder.

### RNA secondary structure prediction and drawing

RNA secondary structures were predicted using mfold through (URL: http://mfold.bioinfo.rpi.edu/cgi-bin/rna-form1.cgi) [64]. Pictures of RNA secondary structures were drawn using RNAviz [65].

## Supporting Information

Figure S1 Genomic context of the typical TE-MIRs. Stem-loop precursors are yellow and annotated mature miRNAs are red.
(JPG)

Figure S2 More example of the typical TE-MIRs small RNA production. Presented in the same way as in Figure 2B.
(JPG)

Figure S3 Formation of TE-MIR by adjacent cognate TEs with inverted orientation. Presented in the same way as in Figure 3.
(JPG)

Figure S4 Examples of site-specific cleavages induced by TE-MIR sRNAs detected by CleaveLand using high-throughput sequencing degradome data. Pairings of the sRNAs and corresponding target sites are indicated by “|”. The positions of cleavage supported by degradome data are indicated by arrows. Accession numbers of degradome databases, category and percent of the abundance in the total reads of the gene are indicated above the arrows and separated by comas. Information of the gene and sRNA is shown at the right side.
(JPG)

Table S1 Plant miRNAs deposited in miRbase that have significant sequence similarity with genomic repeats by BLAST search.
(DOC)

Table S2 Characterization of rice TE-MIRs.
(DOC)

Table S3 Copy number of canonical rice miRNAs in the rice genome.
(XLS)

Table S4 Initial target genes of TE-MIRs predicted to be target of their small RNAs.
(DOC)
