# Supplementary figures and images for: Domestication of Transposable Elements into MicroRNA Genes in Plants
Source: PLoS One. 2011 May 3;6(5):e19212. doi: 10.1371/journal.pone.0019212 (PMC3086885; doi:10.1371/journal.pone.0019212)

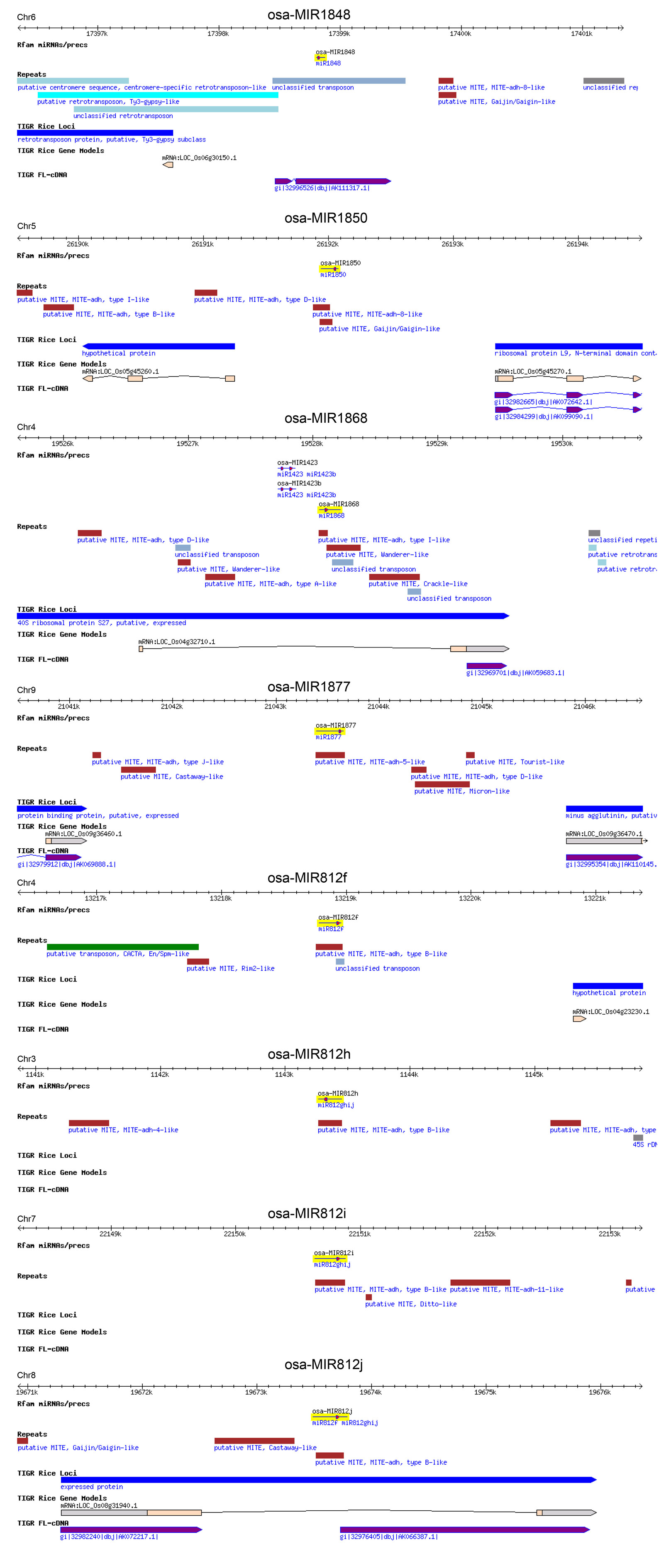

Supplement: Figure S1 — Genomic context of the typical TE-MIRs. Stem-loop precursors are yellow and annotated mature miRNAs are red. (JPG) [file pone.0019212.s001.jpg]

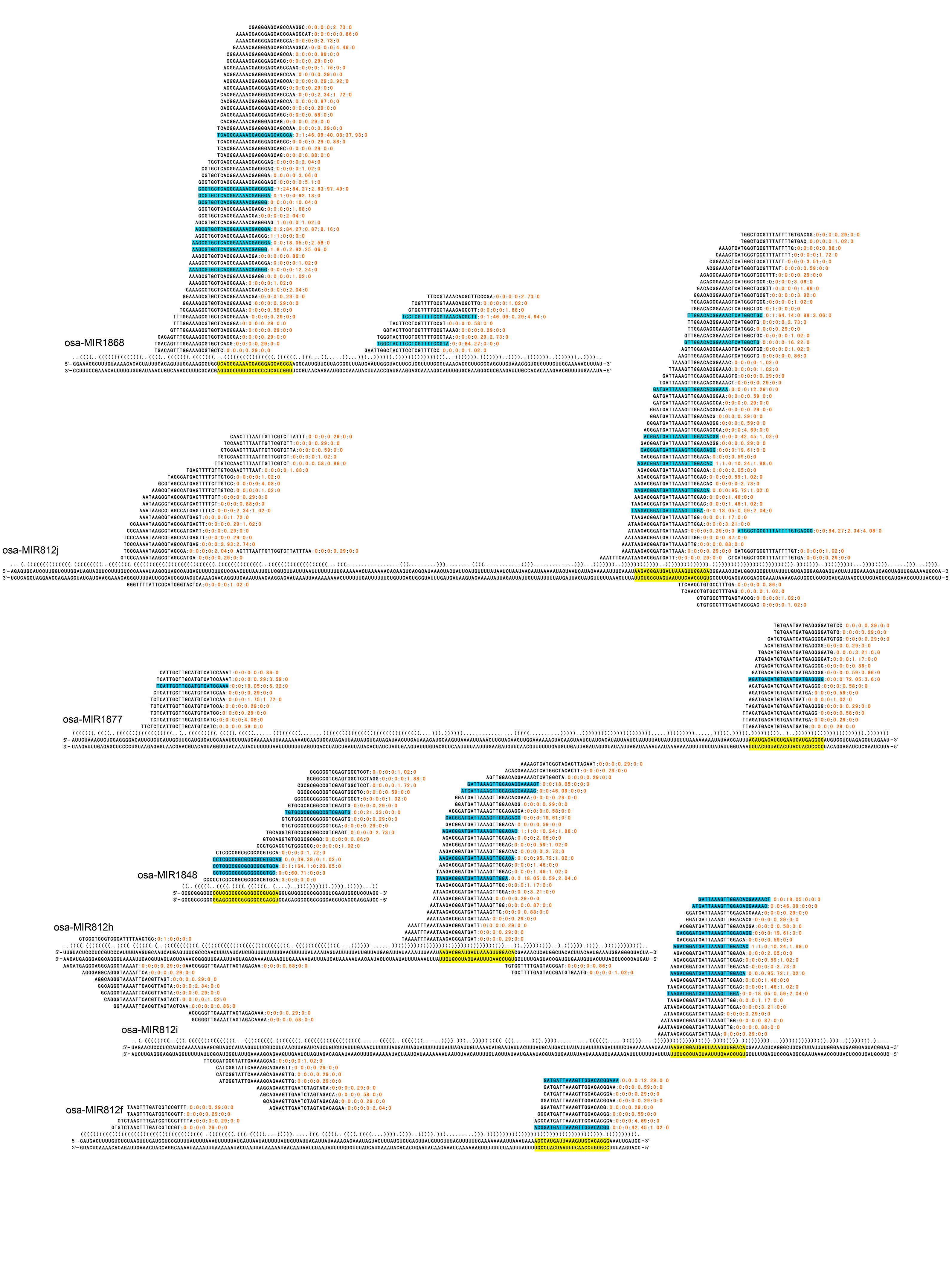

Supplement: Figure S2 — More example of the typical TE-MIRs small RNA production. Presented in the same way as in Figure 2B. (JPG) [file pone.0019212.s002.jpg]

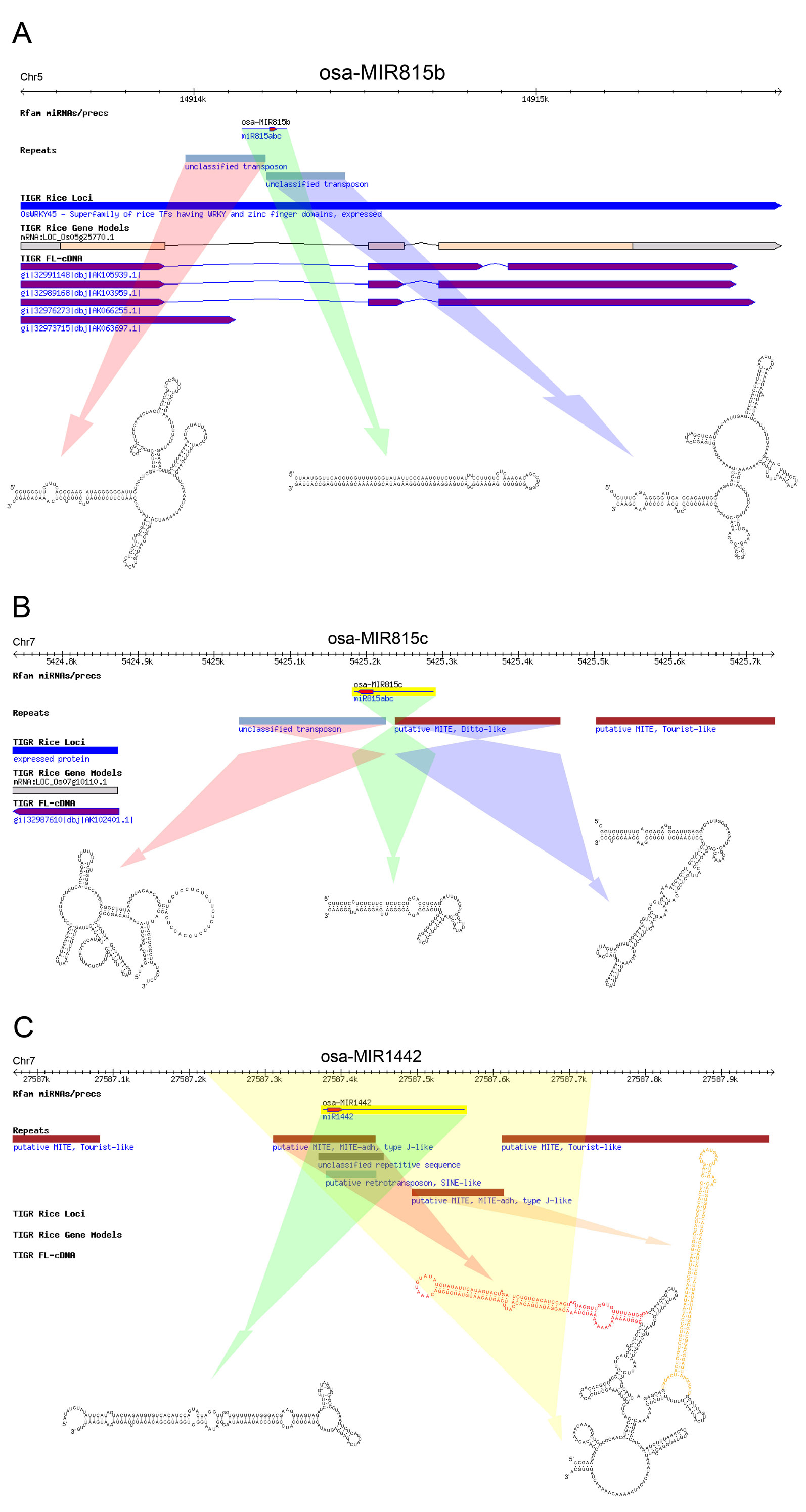

Supplement: Figure S3 — Formation of TE-MIR by adjacent cognate TEs with inverted orientation. Presented in the same way as in Figure 3. (JPG) [file pone.0019212.s003.jpg]

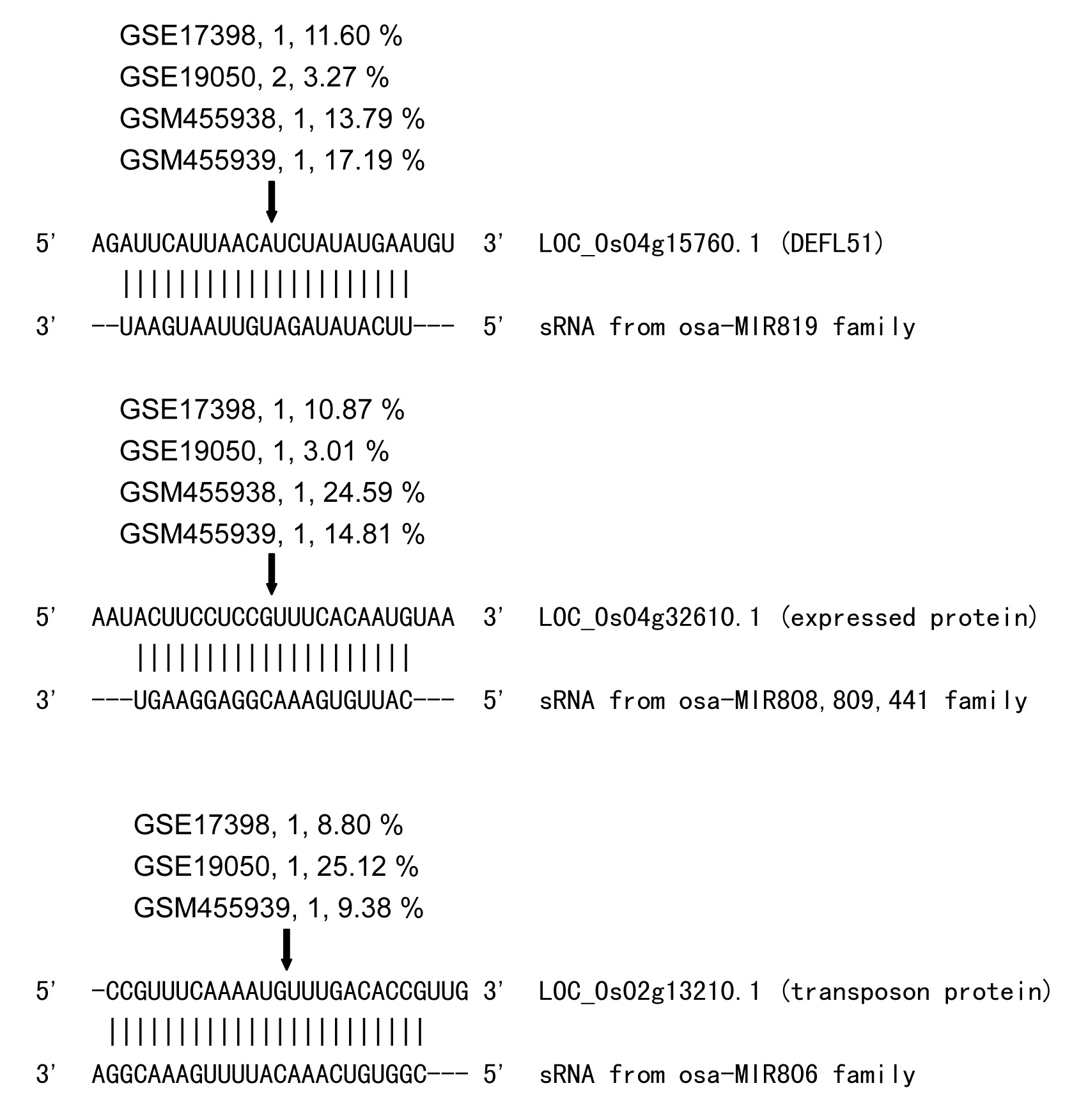

Supplement: Figure S4 — Examples of site-specific cleavages induced by TE-MIR sRNAs detected by CleaveLand using high-throughput sequencing degradome data. Pairings of the sRNAs and corresponding target sites are indicated by “|”. The positions of cleavage supported by degradome data are indicated by arrows. Accession numbers of degradome databases, category and percent of the abundance in the total reads of the gene are indicated above the arrows and separated by comas. Information of the gene and sRNA is shown at the right side. (JPG) [file pone.0019212.s004.jpg]
