# Supplementary material for: Domestication of Transposable Elements into MicroRNA Genes in Plants
Source: PLoS One. 2011 May 3;6(5):e19212. doi: 10.1371/journal.pone.0019212 (PMC3086885; doi:10.1371/journal.pone.0019212)
Supplement: Table S1 — Plant miRNAs deposited in miRbase that have significant sequence similarity with genomic repeats by BLAST search. (DOC) [file pone.0019212.s005.doc]

| **Table S1**. Plant miRNAs deposited in miRbase that have significant sequence similarity with genomic repeats by BLAST search. | | | | | | | |
| --- | --- | --- | --- | --- | --- | --- | --- |
| Classification^a^ | MIR^b^ | Repeat^c^ | S^d^ | Expect^e^ | L^f^ | Range^g^ | Id^h^ |
| CCS1 | ath-MIR401^i^ | ARSiCMCMOOT00007 | + | 2.00E-022 | 250 | 194-247 | 98 |
| unclassified repeat | ath-MIR855^i^ | ARSgOTOT00000017 | - | 3.00E-006 | 272 | 64-98 | 91 |
| MITE-adh-5-like | osa-MIR1877 | ORSgTEMT03000001 | - | 3.00E-015 | 261 | 82-145 | 90 |
| MITE-adh-8 | osa-MIR1850 | ORSiTEMT03300001 | + | 2.00E-005 | 133 | 100-126 | 96 |
| MITE-adh-9-like | osa-MIR811a^i^ | ORSgTEMT03400028 | - | 2.00E-077 | 190 | 3-190 | 94 |
|  | osa-MIR811b^i^ | ORSgTEMT03400037 | + | 1.00E-105 | 190 | 1-190 | 100 |
|  | osa-MIR811c^i^ | ORSgTEMT03400028 | + | 3.00E-063 | 159 | 1-159 | 94 |
| MITE-adh. type B | osa-MIR1862a | ORSiTEMT01600008 | + | 4.00E-005 | 88 | 66-87 | 100 |
|  | osa-MIR1862b | ORSiTEMT01600008 | + | 0.002 | 199 | 143-162 | 100 |
|  | osa-MIR1862c | ORSiTEMT01600008 | - | 1.00E-005 | 89 | 1-23 | 100 |
|  | osa-MIR1862d | ORSiTEMT01600008 | + | 8.00E-005 | 150 | 101-122 | 100 |
|  | osa-MIR1862e | ORSiTEMT01600008 | + | 5.00E-005 | 93 | 70-91 | 100 |
|  | osa-MIR812j | ORSiTEMT01600008 | + | 6.00E-029 | 297 | 125-207 | 93 |
|  | osa-MIR818a | ORSiTEMT01600009 | + | 5.00E-009 | 144 | 111-139 | 100 |
|  | osa-MIR818b^i^ | ORSiTEMT01600009 | + | 6.00E-008 | 113 | 86-112 | 100 |
|  | osa-MIR818c | ORSiTEMT01600009 | + | 4.00E-007 | 176 | 139-164 | 100 |
|  | osa-MIR818d | ORSiTEMT01600009 | - | 2.00E-008 | 139 | 15-46 | 96 |
|  | osa-MIR818e^i^ | ORSiTEMT01600009 | - | 6.00E-016 | 225 | 3-55 | 94 |
| MITE-adh. type B-like | osa-MIR806a | ORSgTEMT01600927 | + | 1.00E-091 | 252 | 9-246 | 92 |
|  | osa-MIR806b^i^ | ORSgTEMT01601752 | + | 1.00E-066 | 215 | 1-215 | 88 |
|  | osa-MIR806c | ORSgTEMT01602539 | + | 3.00E-098 | 270 | 18-256 | 93 |
|  | osa-MIR806d | ORSgTEMT01601807 | - | 7.00E-093 | 271 | 17-254 | 92 |
|  | osa-MIR806e | ORSgTEMT01600979 | + | 1.00E-051 | 255 | 142-246 | 99 |
|  | osa-MIR806f | ORSgTEMT01601372 | - | 1.00E-134 | 241 | 2-240 | 100 |
|  | osa-MIR806g^i^ | ORSgTEMT01601807 | - | 1.00E-094 | 240 | 3-240 | 92 |
|  | osa-MIR806h | ORSgTEMT01600306 | - | 1.00E-134 | 271 | 14-252 | 100 |
|  | osa-MIR812a^i^ | ORSgTEMT01601603 | - | 1.00E-075 | 234 | 1-234 | 89 |
|  | osa-MIR812b^i^ | ORSgTEMT01601516 | - | 2.00E-043 | 170 | 13-169 | 88 |
|  | osa-MIR812e^i^ | ORSgTEMT01602364 | + | 2.00E-024 | 170 | 48-167 | 85 |
|  | osa-MIR812g | ORSgTEMT01601675 | - | 2.00E-012 | 242 | 113-155 | 95 |
|  | osa-MIR812h | ORSgTEMT01600940 | + | 1.00E-010 | 187 | 74-113 | 95 |
|  | osa-MIR1884a | ORSgTEMT01602725 | + | 7.00E-034 | 214 | 103-209 | 91 |
|  | osa-MIR1884b | ORSgTEMT01601805 | - | 9.00E-018 | 221 | 156-211 | 94 |
| MITE-adh. type D-like | osa-MIR441a | ORSgTEMT01702344 | + | 3.00E-063 | 153 | 23-142 | 100 |
|  | osa-MIR441b | ORSgTEMT01701161 | - | 5.00E-034 | 152 | 16-142 | 89 |
|  | osa-MIR441c | ORSgTEMT01702105 | + | 1.00E-083 | 154 | 1-154 | 100 |
|  | osa-MIR446 | ORSgTEMT01700205 | - | 2.00E-083 | 175 | 8-161 | 100 |
|  | osa-MIR808 | ORSgTEMT01701615 | + | 2.00E-082 | 152 | 1-152 | 100 |
|  | osa-MIR809a | ORSgTEMT01701876 | - | 1.00E-034 | 161 | 87-158 | 100 |
|  | osa-MIR809b | ORSgTEMT01700544 | - | 3.00E-044 | 152 | 20-145 | 92 |
|  | osa-MIR809c | ORSgTEMT01700749 | + | 4.00E-025 | 155 | 99-154 | 100 |
|  | osa-MIR809d | ORSgTEMT01700375 | + | 5.00E-074 | 167 | 9-162 | 97 |
|  | osa-MIR809e | ORSgTEMT01701631 | + | 1.00E-087 | 161 | 1-161 | 100 |
|  | osa-MIR809f | ORSgTEMT01700395 | + | 1.00E-083 | 154 | 1-154 | 100 |
|  | osa-MIR809g | ORSgTEMT01700066 | + | 5.00E-034 | 152 | 82-152 | 100 |
|  | osa-MIR809h^i^ | ORSgTEMT01702048 | + | 1.00E-071 | 134 | 1-134 | 100 |
|  | osa-MIR819a^i^ | ORSgTEMT01700791 | - | 4.00E-037 | 125 | 17-123 | 93 |
|  | osa-MIR819b | ORSgTEMT01701364 | + | 2.00E-083 | 166 | 7-160 | 100 |
|  | osa-MIR819c | ORSgTEMT01700540 | - | 1.00E-083 | 154 | 1-154 | 100 |
|  | osa-MIR819d^i^ | ORSgTEMT01700390 | + | 4.00E-059 | 152 | 2-151 | 93 |
|  | osa-MIR819e | ORSgTEMT01701149 | - | 1.00E-083 | 154 | 1-154 | 100 |
|  | osa-MIR819f^i^ | ORSgTEMT01700856 | + | 1.00E-065 | 144 | 1-144 | 96 |
|  | osa-MIR819g^i^ | ORSgTEMT01700703 | + | 2.00E-079 | 147 | 1-147 | 100 |
|  | osa-MIR819h^i^ | ORSgTEMT01700821 | - | 7.00E-061 | 155 | 8-155 | 94 |
|  | osa-MIR819i | ORSgTEMT01702078 | + | 2.00E-083 | 198 | 27-180 | 100 |
|  | osa-MIR819j | ORSgTEMT01701151 | + | 2.00E-083 | 175 | 13-166 | 100 |
|  | osa-MIR819k | ORSgTEMT01701567 | + | 1.00E-083 | 156 | 2-155 | 100 |
| MITE. Buhui-like | osa-MIR2100 | ORSgTEMT03800100 | + | 9.00E-008 | 153 | 3-29 | 100 |
| MITE. ECR-like | osa-MIR807a | ORSgTEMT04200009 | - | 3.00E-039 | 252 | 122-252 | 90 |
|  | osa-MIR807b^i^ | ORSgTEMT04200007 | - | 2.00E-057 | 146 | 1-146 | 93 |
|  | osa-MIR807c^i^ | ORSgTEMT04200032 | + | 6.00E-074 | 184 | 11-184 | 94 |
| MITE. Pangrangja-like | osa-MIR1435 | ORSgTEMT06000346 | - | 2.00E-006 | 183 | 95-119 | 100 |
|  | osa-MIR437 | ORSgTEMT06000059 | - | 3.00E-008 | 213 | 136-175 | 92 |
| MITE. Snabo-like | osa-MIR812c^i^ | ORSgTEMT01400007 | - | 4.00E-013 | 164 | 56-103 | 93 |
|  | osa-MIR812d^i^ | ORSgTEMT01400034 | - | 4.00E-019 | 153 | 53-110 | 94 |
|  | osa-MIR812i | ORSgTEMT01400011 | + | 9.00E-009 | 242 | 110-142 | 96 |
| MITE. stowaway | osa-MIR1439 | ORSiTEMT00200004 | + | 2.00E-008 | 172 | 23-66 | 90 |
| MITE. Tourist-like | osa-MIR1441 | ORSgTEMT00101589 | + | 8.00E-024 | 180 | 1-86 | 90 |
|  | osa-MIR442^i^ | ORSgTEMT00101201 | + | 6.00E-093 | 233 | 1-228 | 93 |
|  | osa-MIR445a^i^ | ORSgTEMT00101488 | + | 2.00E-071 | 268 | 16-170 | 95 |
|  | osa-MIR445b | ORSgTEMT00101488 | + | 3.00E-049 | 264 | 27-138 | 97 |
|  | osa-MIR445c | ORSgTEMT00101488 | + | 2.00E-046 | 260 | 25-137 | 93 |
|  | osa-MIR445d | ORSgTEMT00100694 | + | 8.00E-087 | 312 | 10-305 | 89 |
|  | osa-MIR445e | ORSgTEMT00101488 | + | 3.00E-046 | 265 | 27-139 | 93 |
|  | osa-MIR445f | ORSgTEMT00101488 | + | 7.00E-044 | 305 | 48-160 | 92 |
|  | osa-MIR445g | ORSgTEMT00101488 | + | 3.00E-046 | 275 | 32-144 | 93 |
|  | osa-MIR445h | ORSgTEMT00101488 | + | 1.00E-048 | 277 | 29-145 | 94 |
|  | osa-MIR445i | ORSgTEMT00101019 | + | 1.00E-026 | 244 | 136-238 | 89 |
|  | osa-MIR813^i^ | ORSgTEMT00100446 | - | 7.00E-006 | 203 | 118-157 | 90 |
| Wanderer-like MITE | osa-MIR1868 | ORSiTEMT01000008 | + | 6.00E-009 | 169 | 123-163 | 92 |
| LTR-retrotransposon | osa-MIR420^i^ | ORSiCMCM00100019 | - | 3.00E-066 | 155 | 1-153 | 95 |
| retroposon p-SINE2-r2010 | osa-MIR1436 | ORSiTERT00400041 | - | 3.00E-004 | 161 | 124-144 | 100 |
|  | osa-MIR1442 | ORSiTERT00400041 | - | 4.00E-007 | 185 | 20-69 | 88 |
| retrotransposon | osa-MIR1879 | ORSiTERTOOT00351 | + | 2.00E-005 | 150 | 6-36 | 93 |
| TE CACTA. En/Spm-like | osa-MIR821a^i^ | ORSgTETN00200009 | - | 6.00E-007 | 274 | 242-271 | 96 |
|  | osa-MIR821b^i^ | ORSgTETN00200009 | - | 1.00E-008 | 287 | 249-277 | 100 |
|  | osa-MIR821c^i^ | ORSgTETN00200009 | + | 2.00E-006 | 266 | 1-29 | 96 |
| transposon Tnr11 | osa-MIR812f | ORSiTETNOOT00006 | + | 2.00E-006 | 179 | 81-109 | 96 |
| transposon AnacB3 | osa-MIR1848 | ORSiTETNOOT00104 | - | 5.00E-013 | 63 | 1-60 | 87 |
| transposon Tnr3 | osa-MIR817^i^ | ORSiTETN00200001 | + | 0.003 | 83 | 1-83 | 80 |
| unclassified repeat | osa-MIR1426 | ORSgOTOT00000566 | + | 2.00E-023 | 130 | 1-89 | 89 |
| unclassified transposon | osa-MIR815a | ORSgTETNOOT01417 | + | 1.00E-014 | 83 | 46-83 | 100 |
|  | osa-MIR815b | ORSgTETNOOT00199 | + | 4.00E-031 | 130 | 1-66 | 100 |
|  | osa-MIR815c | ORSgTETNOOT02019 | - | 1.00E-015 | 103 | 64-103 | 100 |
| MITE. stowaway | tae-MIR1120 | TRSiTEMT00200010 | + | 2.00E-015 | 119 | 18-82 | 89 |
|  | tae-MIR1121 | TRSiTEMT00200011 | - | 1.00E-019 | 98 | 9-93 | 88 |
|  | tae-MIR1122 | TRSiTEMT00200008 | - | 4.00E-016 | 295 | 182-245 | 90 |
|  | tae-MIR1126 | TRSiTEMT00200013 | - | 8.00E-010 | 164 | 2-90 | 83 |
|  | tae-MIR1127 | TRSiTEMT00200003 | + | 5.00E-008 | 150 | 70-134 | 86 |
|  | tae-MIR1128 | TRSiTEMT00200009 | - | 2.00E-007 | 190 | 83-115 | 93 |
|  | tae-MIR1133 | TRSiTEMT00200002 | + | 5.00E-039 | 180 | 33-150 | 91 |
|  | tae-MIR1135 | TRSiTEMT00200003 | - | 5.00E-013 | 109 | 35-108 | 86 |
|  | tae-MIR1136 | TRSiTEMT00200009 | + | 4.00E-020 | 107 | 7-92 | 88 |
|  | tae-MIR1137 | TRSiTEMT00200001 | - | 3.00E-015 | 148 | 60-113 | 92 |
| transposon. CACTA. | tae-MIR1117 | TRSiTETN00200022 | + | 4.00E-051 | 118 | 1-118 | 96 |
|  | tae-MIR1131 | TRSiTETN00200019 | + | 2.00E-028 | 100 | 1-100 | 91 |
| 1. TIGR Plant Repeat Database annotation of the repeat sequences. 2. miRbase miRNA gene name. 3. Accession number of the repeat sequence in the TIGR Plant Repeat producing the HSP with the lowest E value for an miRNA gene. 4. Strand of the repeat sequence that match the miRNA gene. 5. Expect value of the HSP reported by the BLAST program. 6. Length of the miRNA gene precursor foldback. 7. Region of the HSP on the query miRNA precursor foldback sequences. 8. Percent of the nucleotide identities for the HSP. 9. TE derived miRNA genes also reported by Piriyaponsa et al.[3] | | | | | | | |
