# Supplementary material for: Domestication of Transposable Elements into MicroRNA Genes in Plants
Source: PLoS One. 2011 May 3;6(5):e19212. doi: 10.1371/journal.pone.0019212 (PMC3086885; doi:10.1371/journal.pone.0019212)
Supplement: Table S2 — Characterization of rice TE-MIRs. (DOC) [file pone.0019212.s006.doc]

| **Table S2**. Characterization of rice TE-MIRs | | | | | | |
| --- | --- | --- | --- | --- | --- | --- |
| MIR | N^a^ | Strand^b^ | Hit^c^ | Len dis^d^ | HCDS^e^ | TF^f^ |
| osa-MIR420 | 19 | both | 250 | low | yes | no |
| osa-MIR437 | 4 | plus only | 46 | low | no | null |
| osa-MIR441a | 70 | both | 839 | mixed | yes | yes |
| osa-MIR441b | 74 | both | 906 | mixed | yes | yes |
| osa-MIR441c | 44 | plus major | 620 | mixed | yes | yes |
| osa-MIR442 | 135 | both | 532 | mixed | yes | yes |
| osa-MIR445a | 76 | both | 278 | mixed | yes | yes |
| osa-MIR445b | 63 | both | 273 | mixed | yes | yes |
| osa-MIR445c | 51 | both | 291 | low | yes | yes |
| osa-MIR445d | 25 | both | 320 | low | yes | yes |
| osa-MIR445e | 69 | both | 268 | mixed | yes | yes |
| osa-MIR445f | 78 | both | 239 | mixed | yes | yes |
| osa-MIR445g | 36 | both | 190 | low | yes | yes |
| osa-MIR445h | 72 | both | 250 | mixed | yes | yes |
| osa-MIR445i | 52 | both | 184 | 24 major | yes | yes |
| osa-MIR446 | 52 | both | 599 | 24 major | yes | yes |
| osa-MIR806a | 199 | both | 586 | 24 major | yes | yes |
| osa-MIR806b | 95 | both | 689 | 24 major | yes | yes |
| osa-MIR806c | 225 | both | 634 | 24 major | yes | yes |
| osa-MIR806d | 155 | both | 630 | 24 major | yes | yes |
| osa-MIR806e | 385 | both | 614 | 24 major | yes | yes |
| osa-MIR806f | 260 | both | 697 | mixed | yes | yes |
| osa-MIR806g | 290 | both | 755 | 24 major | yes | yes |
| osa-MIR806h | 255 | both | 749 | 24 major | yes | yes |
| osa-MIR807a | 27 | minus major | 5 | low | yes | no |
| osa-MIR807b | 20 | both | 11 | 24 major | yes | yes |
| osa-MIR807c | 25 | minus major | 12 | 24 major | yes | yes |
| osa-MIR808 | 206 | both | 745 | mixed | yes | yes |
| osa-MIR809a | 58 | both | 869 | 21 major | yes | yes |
| osa-MIR809b | 185 | both | 746 | mixed | yes | yes |
| osa-MIR809c | 18 | both | 752 | mixed | yes | yes |
| osa-MIR809d | 253 | both | 810 | mixed | yes | yes |
| osa-MIR809e | 44 | minus major | 728 | 24 major | yes | yes |
| osa-MIR809f | 28 | both | 632 | other | yes | yes |
| osa-MIR809g | 105 | both | 802 | mixed | yes | yes |
| osa-MIR809h | 71 | both | 727 | 21 major | yes | yes |
| osa-MIR811a | 60 | both | 22 | 24 major | no | null |
| osa-MIR811b | 70 | both | 30 | mixed | no | null |
| osa-MIR811c | 56 | both | 24 | 24 major | no | null |
| osa-MIR812a | 86 | both | 142 | mixed | yes | yes |
| osa-MIR812b | 42 | both | 304 | 24 major | yes | yes |
| osa-MIR812c | 36 | both | 41 | 21 major | yes | yes |
| osa-MIR812d | 14 | both | 48 | low | yes | yes |
| osa-MIR812e | 63 | both | 196 | mixed | yes | yes |
| osa-MIR812f | 12 | plus only | 3.5 | 24 major | no | null |
| osa-MIR812g | 36 | both | 12 | 24 major | yes | no |
| osa-MIR812h | 40 | plus major | 9 | 24 major | yes | yes |
| osa-MIR812i | 29 | plus major | 7 | 24 major | yes | no |
| osa-MIR812j | 77 | plus major | 9 | 24 major | yes | yes |
| osa-MIR813 | 37 | both | 79 | mixed | no | null |
| osa-MIR815a | 34 | both | 70 | 24 major | yes | no |
| osa-MIR815b | 86 | plus major | 61 | mixed | yes | yes |
| osa-MIR815c | 58 | both | 63 | mixed | yes | yes |
| osa-MIR817 | 4 | both | 6 | 24 major | no | null |
| osa-MIR818a | 16 | both | 174 | low | yes | yes |
| osa-MIR818b | 14 | both | 186 | low | yes | yes |
| osa-MIR818c | 15 | both | 99 | 24 major | yes | yes |
| osa-MIR818d | 38 | both | 247 | 24 major | yes | yes |
| osa-MIR818e | 83 | both | 179 | mixed | yes | yes |
| osa-MIR819a | 156 | both | 375 | mixed | yes | yes |
| osa-MIR819b | 90 | both | 301 | 24 major | yes | yes |
| osa-MIR819c | 210 | both | 343 | mixed | yes | yes |
| osa-MIR819d | 143 | both | 389 | mixed | yes | yes |
| osa-MIR819e | 211 | both | 318 | mixed | yes | yes |
| osa-MIR819f | 133 | both | 343 | mixed | yes | yes |
| osa-MIR819g | 106 | both | 464 | mixed | yes | yes |
| osa-MIR819h | 140 | both | 401 | mixed | yes | yes |
| osa-MIR819i | 40 | both | 277 | 24 major | yes | yes |
| osa-MIR819j | 177 | both | 289 | mixed | yes | yes |
| osa-MIR819k | 148 | both | 253 | mixed | yes | yes |
| osa-MIR821a | 17 | both | 167 | low | no | null |
| osa-MIR821b | 6 | minus only | 20 | low | no | null |
| osa-MIR821c | 8 | both | 117 | low | no | null |
| osa-MIR1426 | 4 | both | 47 | low | yes | no |
| osa-MIR1435 | 0 | null | null | null | null | null |
| osa-MIR1436 | 27 | both | 80 | low | yes | yes |
| osa-MIR1439 | 7 | both | 106 | low | yes | yes |
| osa-MIR1441 | 61 | both | 105 | 24 major | yes | yes |
| osa-MIR1442 | 28 | minus major | 27 | 24 major | yes | yes |
| osa-MIR1848 | 17 | plus only | 16 | 21 major | yes | yes |
| osa-MIR1850 | 29 | plus major | 2 | 21 major | no | null |
| osa-MIR1862a | 26 | plus only | 77 | 24 major | yes | yes |
| osa-MIR1862b | 39 | both | 5 | 24 major | yes | no |
| osa-MIR1862c | 39 | plus major | 66 | 24 major | yes | yes |
| osa-MIR1862d | 55 | plus major | 36 | mixed | yes | yes |
| osa-MIR1862e | 26 | plus only | 73 | 24 major | yes | yes |
| osa-MIR1868 | 58 | plus only | 1 | 24 major | yes | yes |
| osa-MIR1877 | 25 | plus only | 1 | 24 major | no | null |
| osa-MIR1879 | 29 | plus major | 2 | 24 major | yes | yes |
| osa-MIR1884a | 46 | both | 514 | 24 major | yes | yes |
| osa-MIR1884b | 58 | plus major | 40 | mixed | yes | yes |
| osa-MIR2100 | 2 | minus only | 2 | low | yes | no |
| 1. Number of small RNAs perfectly aligned to miRNA foldback at both strand. 2. Strand bias of the expression of the sRNAs on each strand. 3. Average number of hit in the Osa1 v6 rice genome of all the small RNAs. 4. Small RNA length-frequency distribution pattern of each miRNA foldback. “mixed”: mixed expression across the length spectrum without clear-cut concentration on a specific length that, in most cases, peaks at both 21 nt and 24 nt; “low”: difficult to determine due to low sequencing frequency; “other” : most abundant species is out of the range from 20 to 24 nt; In very few cases, “24 major” also include 23 nt sRNAs. 5. Whether the miRNA foldback is homologous to CDS sequence of any annotated gene in Osa1 v6 with expect value no greater than 0.01 in BLAST search. 6. Whether any small RNA on the plus strand of the foldback was predicted to target the homologous CDS by TargetFinder at the threshold of score ≤ 4 and MFE ratio ≥ 73. | | | | | | |
