# Supplementary material for: Domestication of Transposable Elements into MicroRNA Genes in Plants
Source: PLoS One. 2011 May 3;6(5):e19212. doi: 10.1371/journal.pone.0019212 (PMC3086885; doi:10.1371/journal.pone.0019212)
Supplement: Table S4 — Initial target genes of TE-MIRs predicted to be target of their small RNAs. (DOC) [file pone.0019212.s008.doc]

| **Table S4.** Initial target genes of TE-MIRs predicted to be target of their small RNAs. | | | |
| --- | --- | --- | --- |
| LOC^a^ | Expect^b^ | Description^c^ | TE related miRNA^d^ |
| Os01g40320.1 | 2.00E-04 | DNA damage-binding protein 1 | 1868 |
| Os01g69120.2/3 | 2.00E-04 | uncharacterized RNA methyltransferase pc1998 | 1439 |
| Os02g23823.2 | 7.00E-11 | helix-loop-helix DNA-binding protein | 812a, 812b, 812c, 812d, 812e, 812j |
| Os02g32400.1 | 2.00E-06 | 50S ribosomal protein L19, chloroplast precursor | 815c |
| Os02g38690.1 | 2.00E-22 | protein phosphatase 2C | 809e, 441b, 809b, 819c, 809h, 808, 819j, 819d, 819a, 819e, 809g, 446, 809d |
| Os03g28120.1 | 8.00E-07 | potassium channel protein | 812h |
| Os03g29190.1 | 1.00E-12 | PDI | 812a |
| Os03g37960.1 | 2.00E-10 | acyl CoA binding protein | 445b, 445f, 445a, 445e, 445h, 1441, 445d, 442 |
| Os04g15760.1 | 4.00E-42 | DEFL51 - Defensin and Defensin-like DEFL family | 809e, 441b, 809b, 819c, 809h, 808, 819j, 819d, 809a, 819k, 819b, 819a, 819i, 819g, 819e, 809g, 446, 819f, 809d, 809f, 819h |
| Os04g27760.1 | 8.00E-10 | terpene synthase | 807c |
| Os04g30230.1 | 4.00E-05 | F-box/LRR-repeat protein 14 | 809b, 809h, 808, 809g, 809d |
| Os04g55760.1 | 4.00E-12 | OsWAK55 - OsWAK receptor-like protein kinase | 445b, 445f, 445a, 445e, 445c, 445h, 442, 445g |
| Os05g09660.1 | 1.00E-04 | HAD superfamily phosphatase | 815b |
| Os05g10310.1 | 0.003 | acid phosphatase | 446 |
| Os06g07660.1 | 1.00E-05 | myb-like DNA-binding domain, SHAQKYF class family protein | 446 |
| Os06g19560.1 | 1.00E-12 | peptide transporter | 815b |
| Os07g01130.1 | 4.00E-11 | pentatricopeptide containing protein | 809e, 441b, 809h, 808, 809g, 809d |
| Os07g41060.1 | 7.00E-16 | dihydroflavonol-4-reductase | 441a, 809e, 441b, 809b, 819c, 809h, 808, 819j, 809a, 819b, 819g, 809g, 446, 809c, 441c, 809d, 809f, 819h |
| Os08g37444.5 | 2.00E-04 | signal recognition particle receptor | 819c, 819j, 819k, 819b, 819i, 819g, 819e |
| Os08g44660.1 | 0.003 | EF hand family protein | 809c |
| Os09g28830.1 | 3.00E-09 | OsSCP42 - Putative Serine Carboxypeptidase homologue | 819c, 819j, 819d, 819k, 819b, 819a, 819i, 819e, 819f |
| Os10g01680.1 | 2.00E-17 | transferase family protein | 806f, 806e, 806h |
| Os10g35500.2 | 3.00E-04 | epoxide hydrolase | 806f, 806e, 806h, 806c |
| Os10g37070.1 | 1.00E-09 | cytochrome P450 | 806f, 806a, 806e, 806g, 806h, 806d, 806b, 806c |
| Os11g36560.1 | 6.00E-04 | zinc finger C3HC4 type family protein | 809b, 809c, 809d |
| Os11g47160.1 | 2.00E-07 | receptor kinase 1 | 818c, 818e, 818d, 1442, 818b, 818a |
| Os11g47269.1 | 3.00E-13 | DEFL44 - Defensin and Defensin-like DEFL family | 806f, 806a, 806e, 806g, 806h, 806c |
| Os12g27760.1 | 2.00E-05 | OsFBX442 - F-box protein | 806a, 806e, 806g, 806h, 806d, 806c |
| Os12g44350.1 | 8.00E-05 | actin | 806f, 806e, 806g, 806h, 806c |
| Os01g09212.1 | 9.00E-10 | retrotransposon protein, Ty1-copia subclass | 1848 |
| Os01g22770.1 | 7.00E-10 | transposon protein | 809b, 809h, 808, 809d |
| Os01g31400.1 | 7.00E-05 | retrotransposon protein | 1862e, 1862a, 1862d, 812a, 1862c |
| Os02g13210.1 | 2.00E-35 | transposable element protein, Transposase_24 | 806f, 441a, 806a, 809b, 806e, 1884a, 806g, 806h, 806d, 806b, 812b, 809d, 806c |
| Os03g01780.1 | 6.00E-04 | transposon protein | 441a, 809b, 809g, 446, 809d, 809f |
| Os04g19480.1 | 5.00E-20 | retrotransposon protein, Ty3-gypsy subclass | 809e, 441b, 809b, 819c, 809h, 808, 819j, 819d, 809a, 819k, 819b, 819a, 819i, 819g, 819e, 819f, 809d, 809f, 819h |
| Os05g15950.1 | 6.00E-04 | retrotransposon protein | 441b, 809b, 809h, 808, 446, 809d |
| Os05g17050.1 | 0.003 | transposon protein, CACTA. En/Spm sub-class | 809g, 441c, 809d, 809f |
| Os05g28110.1 | 3.00E-06 | retrotransposon protein | 1879 |
| Os06g15710.1 | 3.00E-04 | retrotransposon, Ty1-copia subclass | 812a, 1862c |
| Os06g23090.1 | 0.009 | retrotransposon protein | 818d |
| Os08g08390.1 | 3.00E-04 | retrotransposon protein | 445a, 445e, 445c, 445h, 442, 445g |
| Os10g07574.1 | 2.00E-12 | transposon protein | 815b |
| Os10g11280.1 | 3.00E-17 | retrotransposon protein | 815b |
| Os12g12770.1 | 1.00E-21 | transposon protein, CACTA. En/Spm sub-class | 807b, 807c |
| Os12g13010.1 | 0.001 | transposon protein, CACTA. En/Spm sub-class | 806h |
| Os12g27240.1 | 7.00E-12 | transposon protein, CACTA. En/Spm sub-class | 815b |
| Os12g35380.1 | 0.003 | transposon protein, CACTA. En/Spm sub-class | 809c |
| Os02g31830.1 | 0.01 | expressed protein | 809g, 441c, 809d, 809f |
| Os02g56360.1/3 | 5.00E-08 | expressed protein | 809e, 441b, 808, 809g, 809d |
| Os03g31044.1/2 | 0.003 | expressed protein | 1868 |
| Os04g32610.1/2/3 | 6.00E-04 | expressed protein | 808, 446, 809d |
| Os04g45665.1 | 9.00E-28 | expressed protein | 441a, 809e, 441b, 809b, 809h, 808, 819d, 809a, 809g, 446, 809c, 441c, 809d, 809f |
| Os05g14900.1 | 0.003 | expressed protein | 809g, 441c, 809d, 809f |
| Os05g50440.1 | 6.00E-26 | expressed protein | 809e, 441b, 809b, 819c, 809h, 808, 819j, 819d, 819k, 819b, 819a, 819g, 819e, 809g, 446, 819f, 809d, 819h |
| Os06g18980.1 | 7.00E-05 | expressed protein | 806a, 806e, 446, 806g, 806h, 806d |
| Os07g05550.1 | 1.00E-15 | expressed protein | 806f, 806a, 806e, 1884a, 806g, 806h, 806d, 806b, 806c |
| Os08g31910.1 | 3.00E-18 | expressed protein | 809e, 441b, 809b, 809h, 808, 809g, 809d |
| Os08g38620.3 | 0.003 | expressed protein | 819c, 819j, 819k, 819b, 819i, 819e |
| Os09g13440.2 | 3.00E-04 | expressed protein | 1439 |
| Os09g16990.1 | 6.00E-10 | expressed protein | 1436, 818e, 818d, 1442, 818b |
| Os11g09710.1 | 8.00E-36 | expressed protein | 445b, 445f, 445a, 445e, 445c, 445h, 445d, 445g, 445i |
| Os12g01355.1 | 1.00E-22 | expressed protein | 806f, 806a, 806e, 1884a, 806g, 806h, 806d, 806b, 812b, 806c |
| 1. Locus number of annotated rice gene of the Osa1 version 6. 2. Minimal expect value among all HSPs formed by the CDS and the TE related miRNAs by BLAST search. 3. Annotation of the gene. 4. TE related miRNA fodbacks that suffice: 1) able to form HSP with the CDS with an E ≤ 0.05; 2) at least one plus strand small RNA was predicted to target the CDS at standard TargetFinder thresholds (score ≤ 4 and MFE ratio ≥ 73). | | | |
